# The COP9 Signalosome regulates seed germination by facilitating protein degradation of RGL2 and ABI5

**DOI:** 10.1371/journal.pgen.1007237

**Published:** 2018-02-20

**Authors:** Dan Jin, Ming Wu, Bosheng Li, Birte Bücker, Philipp Keil, Shaoman Zhang, Jigang Li, Dingming Kang, Jie Liu, Jie Dong, Xing Wang Deng, Vivian Irish, Ning Wei

**Affiliations:** 1 Department of Molecular, Cellular and Developmental Biology, Yale University, New Haven, Connecticut, United States of America; 2 State Key Laboratory of Plant Physiology and Biochemistry, College of Biological Sciences, China Agricultural University, Beijing, China; 3 MOE Key Laboratory of Crop Heterosis and Utilization, College of Agronomy and Biotechnology, China Agricultural University, Beijing, China; The University of North Carolina at Chapel Hill, UNITED STATES

## Abstract

The control of seed germination and seed dormancy are critical for the successful propagation of plant species, and are important agricultural traits. Seed germination is tightly controlled by the balance of gibberellin (GA) and abscisic acid (ABA), and is influenced by environmental factors. The COP9 Signalosome (CSN) is a conserved multi-subunit protein complex that is best known as a regulator of the Cullin-RING family of ubiquitin E3 ligases (CRLs). Multiple viable mutants of the CSN showed poor germination, except for *csn5b-1*. Detailed analyses showed that *csn1-10* has a stronger seed dormancy, while *csn5a-1* mutants exhibit retarded seed germination in addition to hyperdormancy. Both *csn5a-1* and *csn1-10* plants show defects in the timely removal of the germination inhibitors: RGL2, a repressor of GA signaling, and ABI5, an effector of ABA responses. We provide genetic evidence to demonstrate that the germination phenotype of *csn1-10* is caused by over-accumulation of RGL2, a substrate of the SCF (CRL1) ubiquitin E3 ligase, while the *csn5a-1* phenotype is caused by over-accumulation of RGL2 as well as ABI5. The genetic data are consistent with the hypothesis that CSN5A regulates ABI5 by a mechanism that may not involve CSN1. Transcriptome analyses suggest that *CSN1* has a more prominent role than *CSN5A* during seed maturation, but *CSN5A* plays a more important role than *CSN1* during seed germination, further supporting the functional distinction of these two *CSN* genes. Our study delineates the molecular targets of the CSN complex in seed germination, and reveals that CSN5 has additional functions in regulating ABI5, thus the ABA signaling pathway.

## Author summary

The control of seed germination and seed dormancy are critical for successful propagation of plant species, and manipulation of these processes is important for agriculture. The COP9 Signalosome (CSN) is a multi-subunit protein complex that regulates proteasome-mediated protein degradation in part as a regulator of SCF ubiquitin E3 ligases. The CSN is important for timely germination of seeds, but its molecular targets in this process is unclear. In this study, we demonstrate that the CSN regulates protein stabilities of two different targets from two antagonistic hormonal pathways, RGL2 of the GA pathway and ABI5 of the ABA pathway. Our genetic and transcriptome analyses showed that, although *csn1-10* and *csn5a-1* exhibit similar defects in timely germination, the mechanisms of how the mutations affect seed germination differ. Since RGL2 is known to be targeted by SCF during germination, the defect in the timely degradation of RGL2 in *csn1-10* and *csn5a-1* is consistent with the role of CSN as a regulator of the SCF. In addition, we show that CSN5A, but not CSN1, has an additional function in regulating ABI5, a downstream inhibitor of germination.

## Introduction

Seed germination launches the active growth phase of a plant, while seed dormancy prevents germination even under optimal growth conditions. The decision and the processes of seed germination are modulated by many factors but predominantly by gibberellin (GA) and abscisic acid (ABA), two phytohormones which act antagonistically on seed germination [1, 2]. ABA levels become elevated during seed maturation to establish and maintain seed dormancy, and its levels drop sharply upon imbibition of seeds. On the other hand, GA biosynthesis starts upon seed imbibition, and GA is necessary to release seed dormancy and stimulate germination [3]. In Arabidopsis, the GA biosynthetic mutant *ga1-3* cannot germinate without an exogenous supply of GA, demonstrating the necessity of GA in seed germination [4, 5]. Various environmental factors such as light, moisture, temperature, and nutrients (e.g. nitrate) can affect germination both during seed maturation and during seed imbibition. Those environmental factors modulate germination in a large part through altering the levels of GA and ABA [6–8]. In the laboratory, seed dormancy is released by a period of dry storage (termed after-ripening) or by cold stratification.

The GA response pathway is negatively controlled by the DELLA proteins, consisting of five members in Arabidopsis: *GA-INSENSITIVE (GAI)*, *REPRESSOR OF ga1-3 (RGA)*, *RGA-LIKE1 (RGL1)*, *RGA-LIKE2 (RGL2)*, and *RGA-LIKE3 (RGL3)* [9]. In response to GA, the DELLA proteins are rapidly degraded by the ubiquitin-proteasome system via SCF^SLY1/2^, which results in GA-stimulated growth and development [10, 11]. Among the DELLA proteins, RGL2 plays a major role as a GA-regulated repressor in seed germination, as *rgl2* can rescue the germination defect of *ga1-3* in the absence of exogenous GA [12, 13]. In addition, RGA and GAI, together with PIL5/PIF1 regulate light-mediated control of seed germination [14, 15]. Under white light, RGL2 plays a predominant role in endosperm tissue, and it also has a central function in the crosstalk with ABA signaling during seed germination [16–18]. ABA induces a number of effectors, including the bZIP transcription factor ABA INSENSITIVE5 (ABI5). ABI5 accumulates during seed maturation and in dry seeds [19, 20]. During the normal course of seed germination, ABA and concomitantly ABI5 levels rapidly decline following imbibition and GA biosynthesis, enabling seed germination. ABI5 has been implicated as the final inhibitor of seed germination, possibly acting downstream of the GA repressor RGL2 [16, 17].

The COP9 signalosome (CSN) is a conserved heteromeric protein complex known to regulate the CULLIN-RING family of ubiquitin E3 ligases (CRLs), including the SCF sub-family of E3s [21]. Biochemically, CSN inhibits CRL E3 activity by removing the NEDD8 (RUB1) modification on the CULLIN subunit (a process known as de-neddylation or de-rubylation) [22, 23], and by direct interaction with the CRL core components [24–26]. However, genetic studies in several organisms including Arabidopsis have shown that CSN promotes the functions of the CRLs [22, 27]. In a number of cases, CSN activity has been shown to protect components of the CRL E3s against their autoubiquitination activity [28, 29]. Still, several studies also indicate that not all substrates of CRL and SCF E3s are regulated by CSN, since some of the SCF substrates display normal signal dependent degradation in CSN-deficient cells [30, 31]. Our understanding of the specific roles of the CSN in SCF-mediated substrate ubiquitination remains incomplete.

In Arabidopsis where the CSN was initially identified, complete loss of any one subunit destabilizes the entire complex [32]. As a result, all of the null *csn* mutants exhibit characteristic purple seeds (the *fusca* phenotype) and developmental arrest soon after germination [33, 34]. Since CSN5 and CSN6 are each encoded by two functionally redundant genes, *CSN5A* vs. *CSN5B*, and *CSN6A* vs. *CSN6B*, respectively, a null mutation in either of the *CSN5* or *CSN6* genes are viable, while knocking out both genes of either *CSN5* (*csn5a-1 5b-1*) or *CSN6* (*csn6a-1 6b-1*) lead to the lethal *fusca* phenotype, like that of other *csn* null mutants [35–37]. Studies using both lethal and weak mutants of the CSN have shown that the CSN is involved in multifaceted developmental processes and physiological responses. For examples, CSN works with SCF^TIR1^ in auxin responses, with SCF^UFO^ in flower development, and with SCF^COI1^ in JA responses. CSN also affects GA signaling, cell divisions, stem cell functions, root patterning, and defenses [22, 27, 38–42]. However, in many of these cases, the specific substrates of the SCF for the corresponding process have not been clearly identified.

It has been observed that lethal mutants of the CSN require extended cold stratification to germinate. The precise germination rates of the lethal mutants were difficult to measure, because the mutants can only be maintained as a heterozygous population. Weak mutants such as *csn5a-2* also show defects in germination [39, 43]. However, the specific targets and the mechanisms underlying this phenotype remain obscure. In this study, we carried out a systematic study of the germination and dormancy phenotypes using viable *csn* mutants, including *csn5a-1* and *csn1-10*. We demonstrate here that CSN regulates seed germination by modulating the levels of RGL2 and ABI5 in the GA and ABA pathways, respectively.

## Results

### Molecular characterization of viable mutants of the CSN

**Fig 1:**
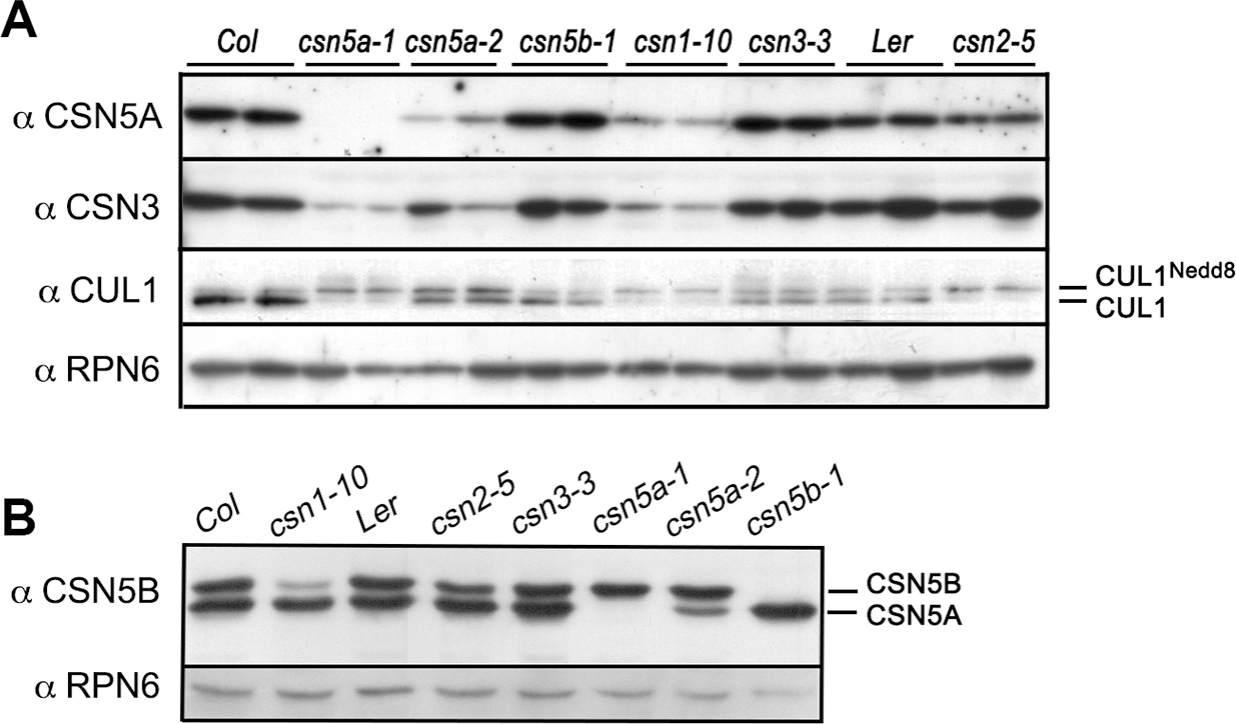
Expression of CSN gene products and CUL1 modification status in various viable *csn* mutants. **(A)** Apical apex tissues from indicated mutants were extracted and the proteins examined by immunoblotting using indicated antibodies. Lower levels of the CSN (CSN3 and CSN5A) and hyper-neddylation of CUL1 were found in *csn5a-1*, *csn5a-2*, *csn1-10*, and *csn2-5*. **(B)** Light-grown seedlings (4-day) of indicated genotypes were used for immunoblotting with anti-CSN5B. The antibody strongly reacted with CSN5B, but also cross-reacted with CSN5A. Endogenous CSN5B migrated slower than CSN5A on the SDS-PAGE. See also S1 Fig.

In recent years, the availability of viable and fertile mutants of Arabidopsis CSN, which can produce homozygous mutant seeds, has provided a feasible genetic material for a systematic analysis of the role of the CSN in seed germination. First, we conducted a basic molecular comparison of a number of viable *csn* mutants for the levels of CSN subunits in plants (**Fig 1 and S1 Fig**). *csn5a-1* and *csn5b-1* are null mutants of the respective genes, while *csn5a-2* is a weak allele of *CSN5A* [37, 44]. The *CSN5A* gene is considered to predominate over *CSN5B*, based on multiple microarray datasets [36] as well as the observation that *csn5a-1* mutants showed a more severe growth defect than those of *csn5b-1* mutants [35, 37] (**S1A Fig**). The anti-CSN5A antibody could readily detect endogenous CSN5A, but not CSN5B (**Fig 1A** and **S1B Fig**), while the anti-CSN5B antibody could detect both CSN5A and CSN5B, and showed that endogenous CSN5B proteins migrate more slowly than CSN5A in SDS-PAGE (**Fig 1B**). It should be mentioned that under our growth conditions, *csn5a-2* plants can tolerate a considerable reduction of CSN5A levels (**S1B Fig**), without showing obvious growth defects, although the same *csn5a-2* allele was previously reported to show a noticeable growth defect [35, 37]. For the other viable mutants used, *csn1-10*, which contains a point mutation that reduces the level of *CSN1* expression [45], exhibited a clear growth defect, while *csn3-3* [46] and *csn2-5* [*Landsberg erecta* (L*er*) background] [47] appeared to be phenotypically similar to the respective wild type plants (**S1A Fig**).

In Arabidopsis, CSN subunits are stabilized through assembly of the complex [32, 35, 48]. Immunoblot analyses showed that this group of viable *csn* mutants contained variably lowered protein levels of other CSN subunits (**Fig 1** and **S1 Fig**). In particular, *csn5a-1* and *csn1-10* displayed the most noticeable reductions in the steady state level of CSN3 and CSN5 (**Fig 1**), suggesting that the level of the CSN complex was lower in these mutants. Consistent with previous reports, *csn5a-1*, *csn5a-2*, *csn1-10*, and *csn2-5*, but not *csn5b-1* or *csn3-3*, caused hyperneddylation of CUL1, indicating that these mutants had lower CSN-mediated deneddylation activity (**Fig 1**). Therefore, *csn5a-1*, *csn5a-2*, *csn1-10*, and *csn2-5* are definitively partial loss-of-function mutants of the CSN.

### *csn* mutants exhibit hyperdormancy and delayed seed germination

**Fig 2:**
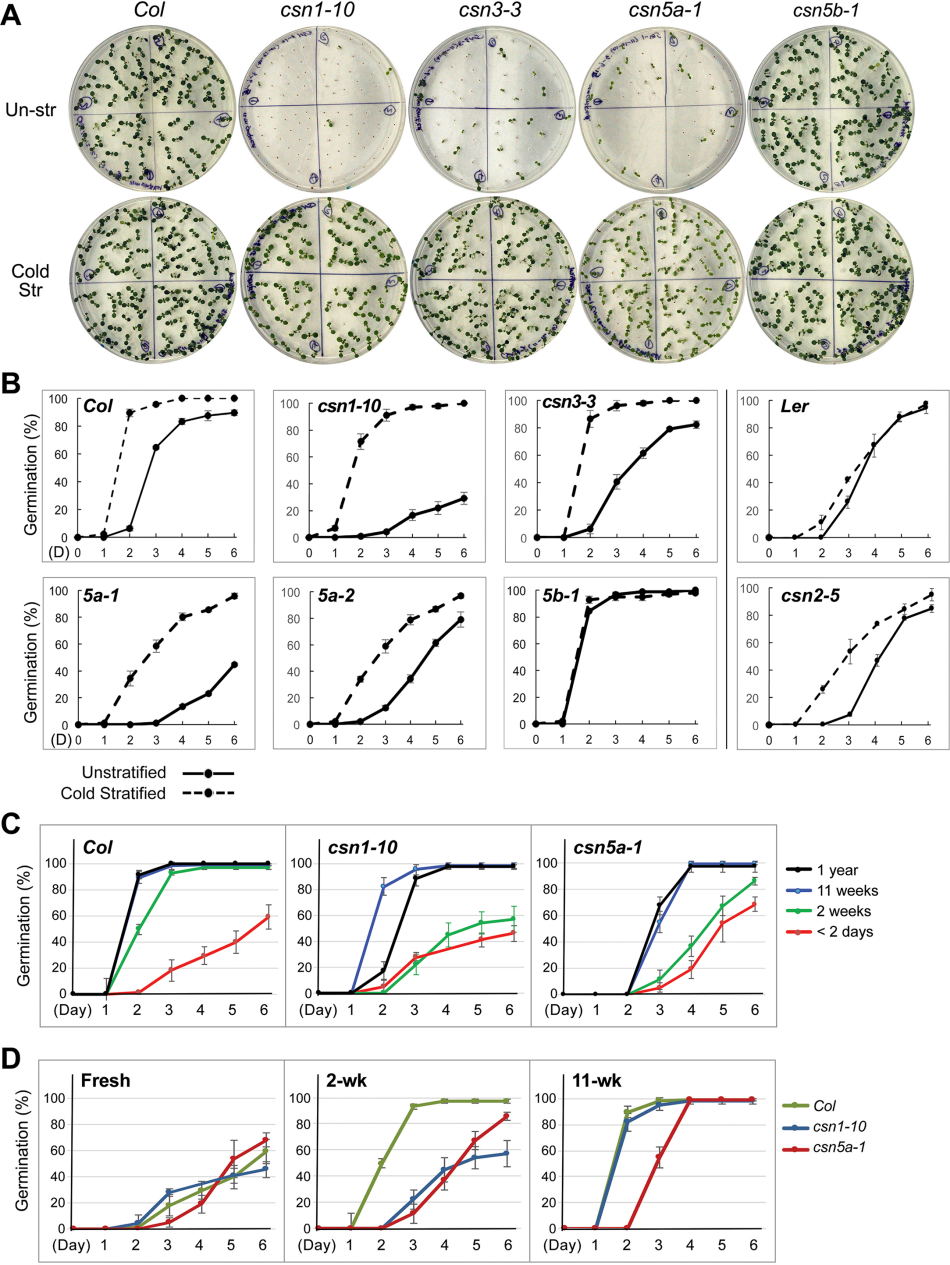
Seed germination phenotype of the *csn* mutants. **(A)** Representative images showing seed germination status of indicated mutants. Seeds were cold stratified for 4 days (Cold Str) or had not stratified (Un-str). Photographs were taken at day 6. **(B)** Germination rates of unstratified (solid lines) or cold stratified (broken lines) seeds of the *csn* mutants. *csn5a-1*, *csn5a-2*, *csn5b-1*, *csn1-10*, *csn3-3* were compared to *Col* wild type, while *csn2-5* to *Ler* wild type. With the exception of *csn5b-1*, all other *csn* mutants tested exhibited slower and lower germination rates compared to *Col*, when not cold stratified. **(C)** Germination rates of *Col*, *csn1-10* and *csn5a-1* seeds that had been in dry storage for indicated period of time. *csn1-10* and *csn5a-1* seeds required extended after-ripening time than *Col* to release dormancy. *csn5a-1* additionally showed delayed germination. **(D)** Germination rates of freshly collected, partially after-ripened (2 weeks), or fully after-ripened (11 weeks) seeds of *Col*, *csn1-10*, and *csn5a-1*. The germination deficiency of *csn1-10* and *csn5a-1* were most readily observed with the seeds of two weeks after seed-collection. *csn5a-1* seeds exhibited a delay in germination even in fully after-ripened seeds. In all panels, error bars represent standard deviation from 4 repeats (n = 4). See also S2 and S3 Figs.

We examined germination of the *csn* mutant seeds with or without cold stratification. When Arabidopsis Columbia (*Col*) seeds were cold stratified at 4°C for 3 days or more, germination was accelerated by 12–24 hours over unstratified seeds under our growth and planting conditions (**Fig 2A and 2B**), indicating that those *Col* (wild type) seeds exhibited a weak dormancy response. Without cold stratification, *csn1-10*, *csn3-3*, *5a-1* and *5a-2* exhibited noticeably delayed and poor germination compared to Col. *csn2-5* also exhibited a slight but consistently poor seed germination compared to the corresponding wild type (L*er*). The germination defects could be nearly completely alleviated by cold stratification in *csn1-10*, *csn3-3*, and *csn2-5* seeds (**Fig 2A and 2B**). However, in *csn5a-1* and *csn5a-2*, cold stratification could significantly, but not completely, alleviate their germination defects (**Fig 2B, S2 Fig)**. These observations suggest that most of the *csn* mutants had stronger seed dormancy. The exception was *csn5b-1*, which appeared to have a weaker dormancy than *Col*, as it germinated equally well regardless whether or not the seeds had been cold stratified. Since our medium contained 1% sucrose, we tested mutant germination on plates without sucrose, and found that *csn1-10* and *csn5a-1* showed similar germination phenotype regardless of whether sucrose was present or not (**S2A Fig**).

To further confirm the seed dormancy phenotype, we examined the germination rates in relation to different after-ripening storage ages of the seeds in *csn1-10*, *csn5a-1*, and the *Col* control. As shown in **Fig 2C and 2D**, freshly collected seeds (1 or 2 days after collection) of either *Col* or the mutants showed low and heterogeneous germination. While the *Col* seeds showed significant dormancy-release after two weeks, *csn1-10* and *csn5a-1* seeds continued to germinate slowly, consistent with the stronger seed dormancy of the mutants. After extended dry storage (11 weeks), *csn1-10* mutants could catch up with *Col* in germination, but *csn5a-1* still displayed a delay in germination compared to *Col* even in fully after-ripened seeds (1-year) (**Fig 2C and 2D**). This result, as well as the observation that cold stratification could not fully alleviate the germination defect of the *csn5a* mutants, suggest that while *csn1-10* has a seed hyperdormancy phenotype, *csn5a-1* shows a delay in germination in addition to hyperdormancy. As shown in **Fig 2D**, the germination phenotype of the mutants is best displayed in partially after-ripened seeds, typically between 1–4 weeks after seed collection, although the window of phenotypic alteration varies depending on different batches of seeds.

Since most of the *csn* mutants have been reported to have mild photomorphogenic phenotypes, we tested the response of the mutants to phytochrome B (phyB)-controlled seed germination. To distinguish the light response from other aspects of dormancy responses, cold stratified seeds were used. Seeds were then subjected to a 5-min pulse of red (R) light, far red (FR) light, or alternating R and FR treatments in a sequential order as indicated in **S3 Fig**. The *det1-1* mutant, which lacks a phyB-controlled seed germination response and germinates regardless of light treatments [49], was used as a control. The data showed clearly that all of the tested *csn* mutants, including *csn1-10*, *csn3-3*, *csn5a-1* and *csn5b-1*, displayed normal responses in phyB-mediated seed germination (**S3 Fig**). Thus, for the rest of the studies on the mechanisms of the CSN-regulated seed germination, we carried out the experiments under white light. When not specified, un-stratified seeds were used for the germination and biochemical analyses.

### The germination phenotype of *csn1-10* and *csn5a-1* is seed-coat-dependent

The seed coat plays an important role in seed dormancy in many plant species including Arabidopsis [50]. The Arabidopsis seed coat consists of an outer layer of maternally derived material known as the testa. Underneath the testa is a single cell layer of endosperm that encloses the embryo. In the hyperdormant ecotypes *Cvi* and *C24*, or in non-germinating *ga1-3* mutants, removal of the seed coat can break dormancy and allow the embryo to grow [51]. To determine whether the germination defects in *csn1-10* and *5a-1* were imposed by the seed coat, we removed the seed coat by dissection, and observed the growth of the embryos with regard to radicle (embryonic root) growth and cotyledon greening and expansion.

**Fig 3:**
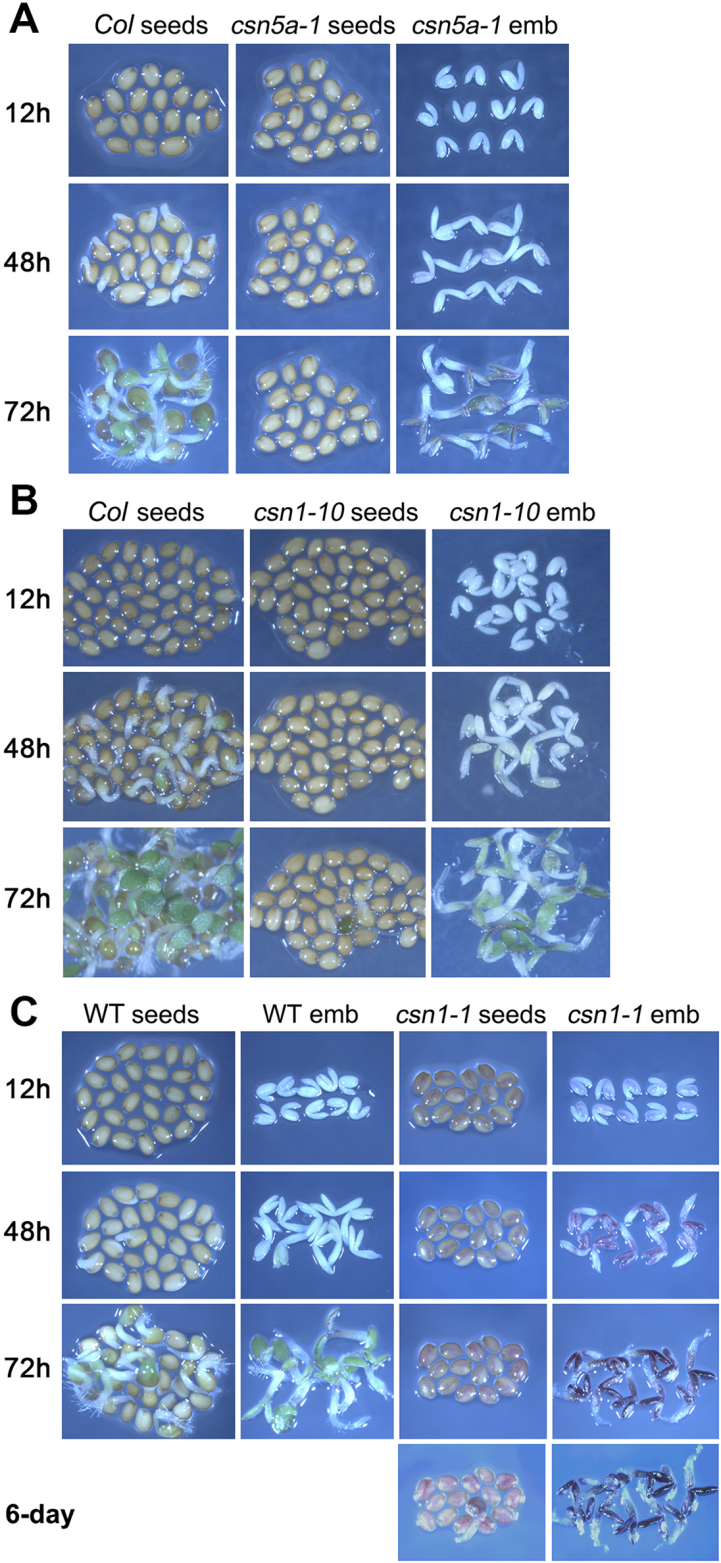
The germination defects of *csn1* and *csn5a* are seed-coat dependent. Intact seeds or embryos dissected from the same seed population were germinated on water-agar plates. **(A)** *csn5a-1*, or **(B)** *csn1-10*, with the corresponding wild type C*ol* seeds as the controls. **(C)** *csn1-1 (fus6-1)* mutant seeds were picked out from the heterozygous (+/-) seed population based on the dark purple seed color, and normal colored seeds were used as the positive control (WT). While the seeds of the *csn* mutant could not germinate on time, their embryos showed normal progression of growth, with radicle elongation on day-2 and cotyledon greening and expansion on day-3. *csn1-1* mutants arrested the growth after germination.

Without the seed coat, both *csn5a-1* (**Fig 3A**) and *csn1-10* (**Fig 3B**) mutant embryos displayed radicle growth on day-2, and exhibited greening and cotyledon expansion on day-3, similar to the developmental time line of comparably treated *Col* embryos and seeds. Remarkably, the removal of the seed-coat even rescued the germination of *csn1-1* (or *fus6-1*, *cop11-1*) (**Fig 3C**), the null mutant of *csn1*, which were otherwise extremely dormant such that mutant seeds rarely germinate without cold stratification. After germination, *csn1-1* mutants arrested further development, and they died as purple seedlings (**Fig 3C** bottom right corner panel), similar to the final morphology of the seedlings germinated from intact seeds [34]. These results showed that *csn1* and *csn5a* mutant embryos have the intrinsic capacity to initiate growth on a similar time scale to the *Col* embryos, and that the hyperdormancy phenotype of the mutant seeds is dependent on the seed coat. Seed coat-dependent inhibition of seed germination has been shown to be mediated at least in part by RGL2 in the endosperm, where it stimulates ABA synthesis, and ultimately ABI5 activity [16, 51].

### *csn* mutants are defective in timely removal of germination inhibitors RGL2 and ABI5

**Fig 4:**
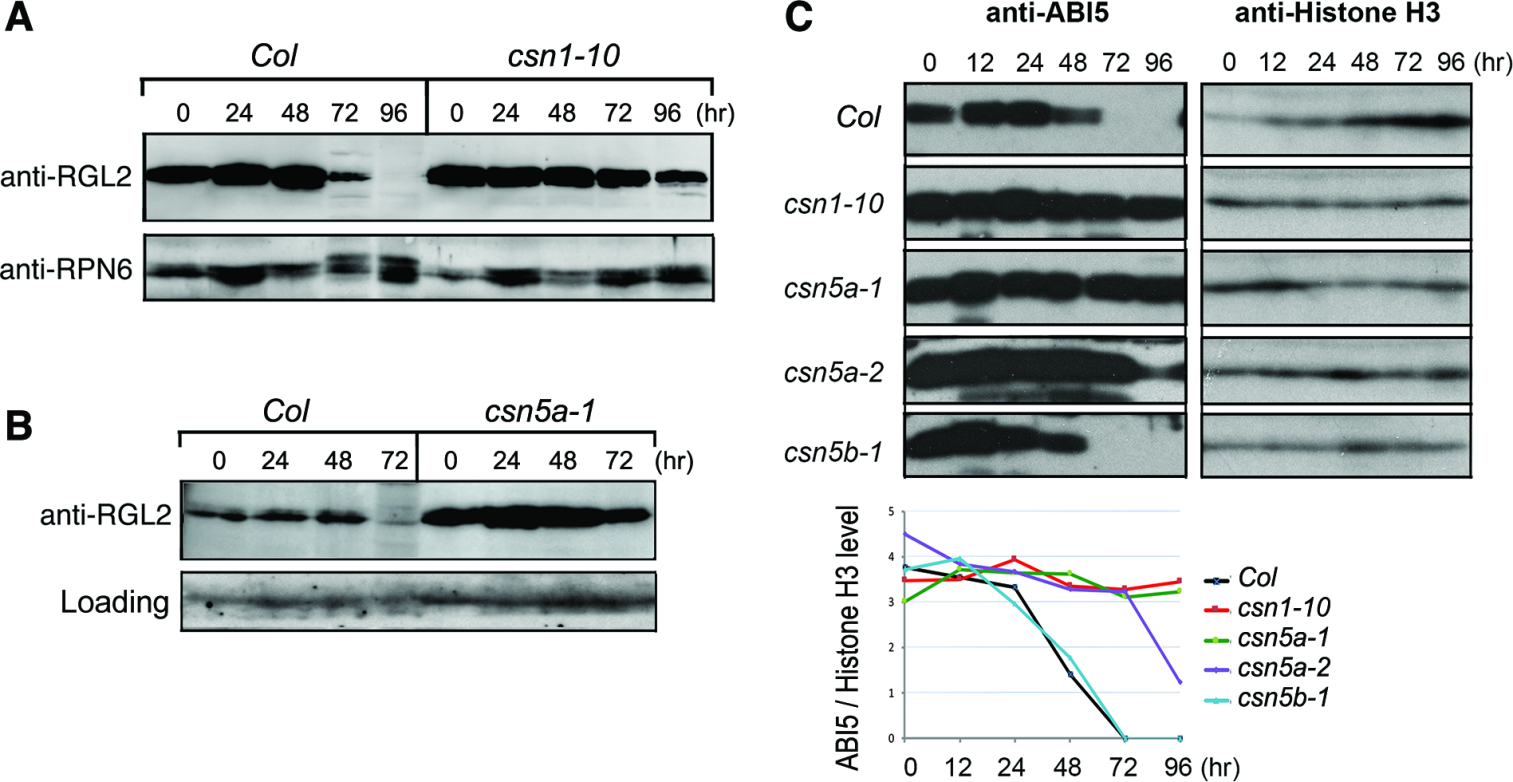
*csn1-10* and *csn5a-1* showed defects in timely down-regulation of RGL2 and ABI5 proteins following seed imbibition. **(A and B)** Time-course analysis of RGL2 levels by immunoblots showing the deficiency of *csn1-10* **(A)** and *csn5a-1* **(B)** in timely decline of RGL2 protein compared to wild type (*Col*). Anti-RPN6 blot or a background band were shown as the loading reference. **(C)** Time course analysis of ABI5 level by immunoblotting. *csn1-10*, *csn5a-1*, *csn5a-2*, but not *csn5b-1*, showed defect in timely decline of ABI5 protein following seed imbibition. Anti-histone H3 was used as an internal loading reference. The lower panel shows the densitometry quantification of ABI5 relative to the corresponding Histone H3 in each sample. Samples were collected at indicated time points in hours (hr) post imbibition, as described in Materials and Methods.

GA stimulate seed germination by removal of GA inhibitors known as DELLA proteins. Among the five DELLA proteins in Arabidopsis, RGA and GAI function mostly in the dark and in the embryos, while RGL2 is the main inhibitor of seed germination under light [12, 18]. RGL2 also plays a central role in seed coat-dependent inhibition of seed germination, which likely involves CSN. RGL2 is predominantly regulated at level of protein turnover via SCF^SLY1^ E3 ligase mediated protein degradation through the ubiquitin-proteasome system [10, 11]. We examined RGL2 protein levels in the *csn* mutants by anti-RGL2 immunoblotting in a time course analysis. Both *csn1-10* (**Fig 4A**) and *csn5a-1* (**Fig 4B**) displayed clear defects in timely degradation of RGL2. Abnormal accumulation of RGL2 is consistent with the idea that degradation of RGL2 via an SCF E3 ubiquitin ligase requires a fully active CSN complex.

Another major inhibitor of seed germination is ABI5, which accumulates in dry seeds and in response to low GA or high ABA conditions. We next examined ABI5 protein levels by anti-ABI5 immunoblotting over the course of germination. While ABI5 proteins were depleted by day-3 in *Col* wild type and *csn5b-1* after imbibition, they remained high in *csn1-10* and *csn5a-1* mutants, and showed slower kinetics of the decline in *csn5a-2* (**Fig 4C**). These results implied that *csn1* and *csn5a* mutants are defective in ABI5 downregulation following imbibition of the seeds. Taken together, *csn1-10* and *csn5a* mutants exhibited defects in the timely removal of RGL2 and ABI5, two key inhibitors of seed germination in Arabidopsis, which correlated with the deficiency of the mutants in their timely germination.

### RGL2 accumulation underlies the germination defect of *csn1-10*, but it is insufficient to account for the germination phenotype of *csn5a-1*

**Fig 5:**
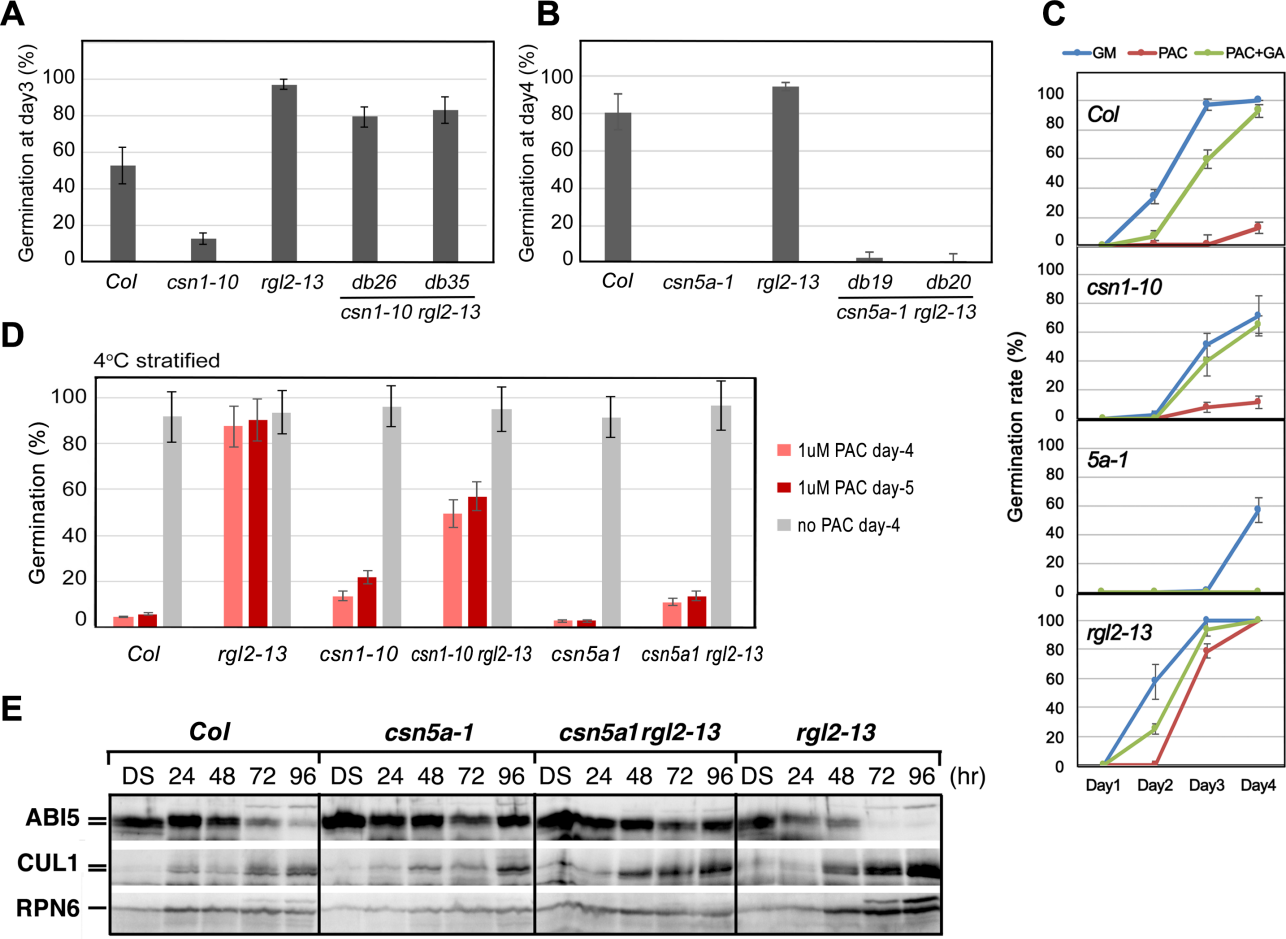
*csn1-10* and *csn5a-1* show different genetic interactions with *rgl2-13*, and different responses to PAC. **(A and B)** *rgl2-13* rescued the germination deficiency of *csn1-10* but not *csn5a-1*. **(A)** The *csn1-10 rgl2-13* double mutants displayed normal germination rate as wild type *Col* and *rgl2-13*, in contrast to low germination of *csn1-10*. Double mutants db26 and db35 are two different lines of the *csn1-10 rgl2-13*. **(B)** Germination rate of *csn5a-1 rgl2-13* double mutants displayed low germination rate similar to *csn5a-1* single mutant, compared to wild type *Col* and *rgl2-13*. Double mutants db19 and db20 are two different lines of the *csn5a-1 rgl2-13*. **(C)** *csn5a-1* was hypersensitive to PAC. Cold stratified seeds were germinated on regular GM plate, GM plate containing PAC (1 μM), or PAC (1 μM) plus GA3 (50 μM). Seed germination rates were shown in graphs. **(D)** *rgl2-13* conferred resistance to PAC (1 μM) in germination of *rgl2-13* and double mutant *csn1-10 rgl2-13*, but not double mutant *csn5a-1 rgl2-13*. **(E)** The *csn5a-1 rgl2-13* double mutant showed abnormally prolonged accumulation of ABI5, similar to *csn5a-1* single mutant. Samples were taken at indicated times after imbibition of the seeds, and proteins were probed with antibodies as indicated on the left. **(A-D)** Error bars represent standard deviation from 4 repeats (n = 4). See also S4 Fig.

If accumulation of RGL2 is responsible for the germination phenotype of the *csn* mutants, genetic removal of the *RGL2* gene should be able to rescue the defects of the *csn* mutants. To test this, we crossed the *csn* mutants with the null mutant of *RGL2*, *rgl2-13 (Col)* [13], and generated the *csn1-10 rgl2-13* and *csn5a-1 rgl2-13* double mutants. Germination tests showed that introduction of *rgl2-13* into the *csn1-10* mutant effectively rescued its germination phenotype (**Fig 5A**). Moreover, *rgl2-13* could significantly alleviate the poor germination of the *csn1-1* lethal allele, enabling the un-stratified *csn1-1 rgl2-13* double mutant seeds to germinate, albeit at a slower rate (**S4 Fig**). In contrast to *csn1*, the germination defect of *csn5a-1* could not be rescued by *rgl2-13* (**Fig 5B**). The *csn5a-1 rgl2-13* double mutant showed a poor germination profile that was similar to that of *csn5a-1* (**Fig 5B**). These results suggest that, while over-accumulation of RGL2 is responsible for the seed hyperdormancy of *csn1-10*, additional components may be involved in the germination defects of *csn5a-1*.

The *csn1-10* and *csn5a-1* mutants also differ in their responses to low GA conditions induced by PAC (paclobutrazol), an inhibitor of GA biosynthesis. To test the sensitivity to PAC, cold stratified seeds were used to factor out the dormancy effect. Treatment with PAC inhibited germination of wild type seeds, and the inhibition could be reversed by simultaneously supplying GA3 (PAC + GA, **Fig 5C**). As previously reported [12, 13], *rgl2-13* was resistant to PAC in germination assays. Both *csn1-10* and *csn5a-1* were sensitive to PAC as *Col*, and the germination of *Col* and *csn1-10*, but not of *csn5a-1*, could be significantly restored by addition of GA3 (**Fig 5C**), indicating that *csn5a-1* was hypersensitive to PAC-induced low GA condition. We next examined the double mutants for their responses to PAC. As shown in **Fig 5D**, *rgl2* and *csn1 rgl2* mutants were able to germinate well in PAC, indicating that removal of RGL2 is sufficient to overcome the effect of PAC even when *CSN1* is deficient. This result is in agreement with the notion that the main function of CSN1 in germination is to modulate RGL2 turnover. However, the *rgl2* mutation had only a slight effect on overcoming PAC treatment in the background of *csn5a-1*, as the germination rates of *csn5a rgl2* double mutants remained very low in PAC (**Fig 5D**), suggesting that removal of *RGL2* was insufficient to allow germination in the absence of *CSN5A*. Thus, it appeared that an additional factor(s) apart from RGL2 was affected by *csn5a-1*, that prevented timely germination or for germinating under low GA conditions. Time-course immunoblotting showed that, as in the *csn5a-1* single mutant, high levels of ABI5 still accumulated in *csn5a-1 rgl2-13* on day-4 post imbibition (**Fig 5E**). This result pointed to ABI5 as a factor that is potentially regulated by CSN5A.

### Loss of ABI5 can rescue the germination phenotype of both *csn1-10* and *csn5a*

**Fig 6:**
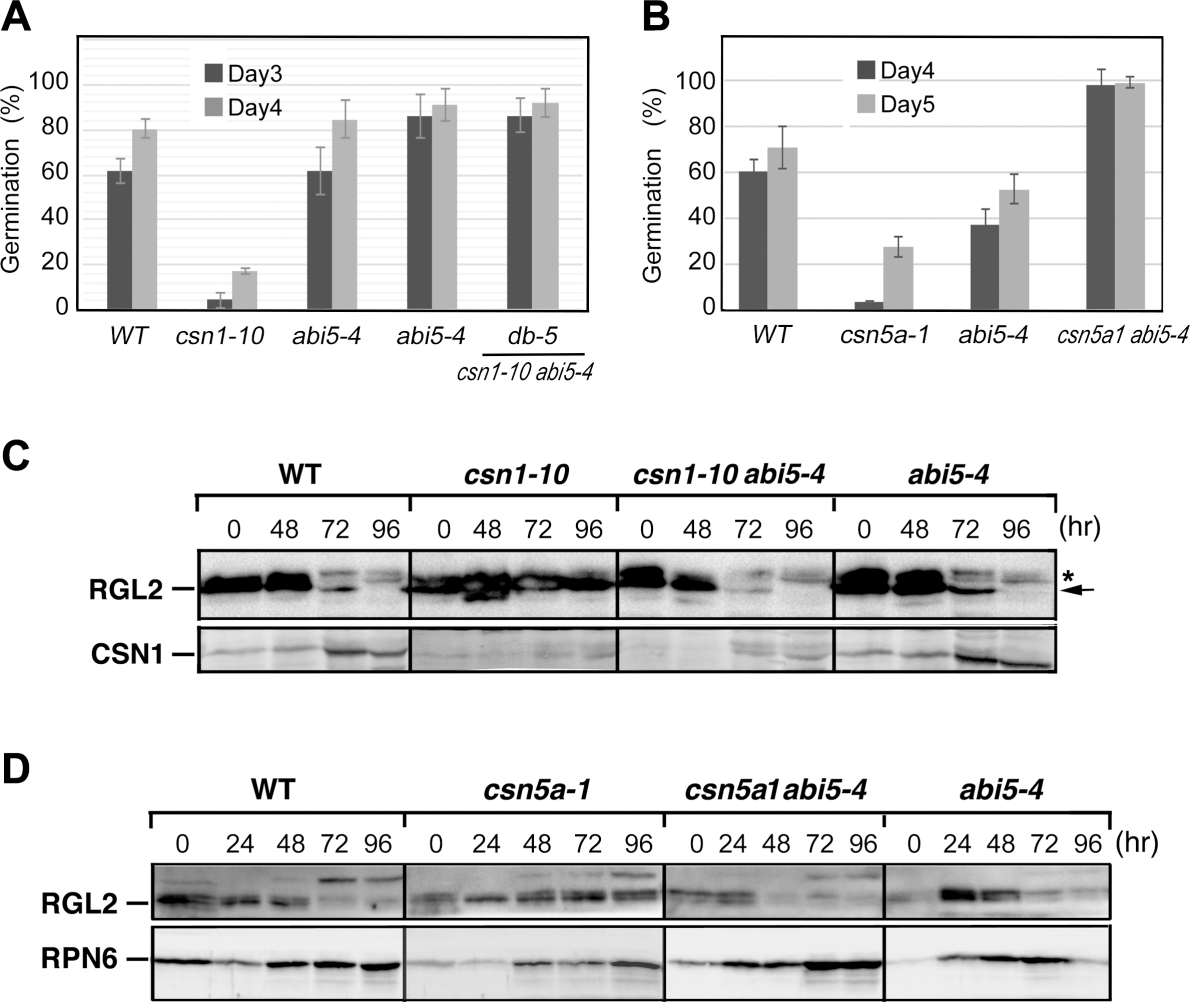
*abi5-4* mutant rescued the germination of *csn1-10* as well as *csn5a-1*. **(A and B)** Germination of *csn1-10 abi5-4* **(A)** or *csn5a-1 abi5-4* **(B)** double mutants displayed normal rate, in contrast to slow germination of the corresponding single mutant *csn1-10* and *csn5a-1*, respectively. All of the *csn1-10 abi5-4* lines including double mutant *db-5*, wild type (WT), *csn1-10*, and *abi5-4* are segregating sibling lines from the same genetic cross. Likewise, all of the *csn5a-1 abi5-4* lines including the double mutant *csn5a-1 abi5-4*, wild type (WT), *csn5a-1*, and *abi5-4* are segregating sibling lines from the same genetic cross. Error bars represent standard deviation from 4 repeats. **(C and D)** Anti-RGL2 immunoblots showing that timely removal of RGL2 was restored in *csn1-10 abi5-4* double mutant **(C)** as well as in *csn5a-1 abi5-4* double mutant **(D)**. Samples of indicated lines were taken at indicated times after seed imbibition. See also S5 Fig.

ABI5 is thought to act downstream of RGL2 to inhibit seed germination under unfavorable environments [16, 17, 52]. To address whether the characteristic over-accumulation of ABI5 protein in the *csn* mutants was responsible for their germination phenotypes, double mutants of *abi5-4* with *csn1-10* or *csn5a* were generated. Since the *abi5-4* mutant is in the Wassilewskija (*Ws*) ecotype [20] while *csn5a-1* and *csn1-10* mutants are in the *Col* background, we used segregating sibling lines of different genotypes in all of the germination comparisons, including *WT*, *csn1-10*, and *csn5a-1*, *abi5-4*, and the double mutants (**Fig 6**), in which “*WT*”was a segregating sibling line with a wild type genotype. Clearly, *abi5-4* rescued the germination phenotype of both *csn1-10* and *csn5a-1*, as demonstrated by strongly improved germination in the respective double mutants, i.e. *csn1-10 abi5-4* (**Fig 6A**), or *csn5a-1 abi5-4* (**Fig 6B**) compared to *csn1-10* or *csn5a-1*, respectively. In addition, the double mutant of *abi5-1 csn5a-2* also rescued the slow germination of *csn5a-2*, a weaker mutant of *csn5a* (**S5 Fig**). The finding that *abi5-4* can rescue the germination phenotype of *csn1-10*, or its over-accumulation of RGL2, is in agreement with the report that the *abi5* mutant can suppress the RGL2-mediated block of germination such as in PAC treatment [16]. These data showed that ABI5 is epistatic to CSN1 in the same pathway during seed germination, and that accumulation of ABI5 is responsible for the germination defects of *csn5a-1*.

Associated with the rescue in germination, time-course immunoblots showed, somewhat surprisingly, that timely removal of RGL2 has also been restored in the double mutants of *csn1-10 abi5-4* as well as *csn5a-1 abi5-4* following germination (**Fig 6C and 6D**). Although the mechanism is unclear as to how RGL2 turnover can be rescued in the absence of ABI5, these results are in agreement with previous reports that low ABI5 is associated with germination in low GA conditions (for example treatment of PAC) [16]. Together, our data further reinforce the notion that ABI5 functions downstream of RGL2 in seed germination, and that proper regulation of both germination inhibitors requires CSN5A.

### Differential impacts of CSN1 and CSN5A on transcriptomes in dry seeds vs the imbibed seeds

**Fig 7:**
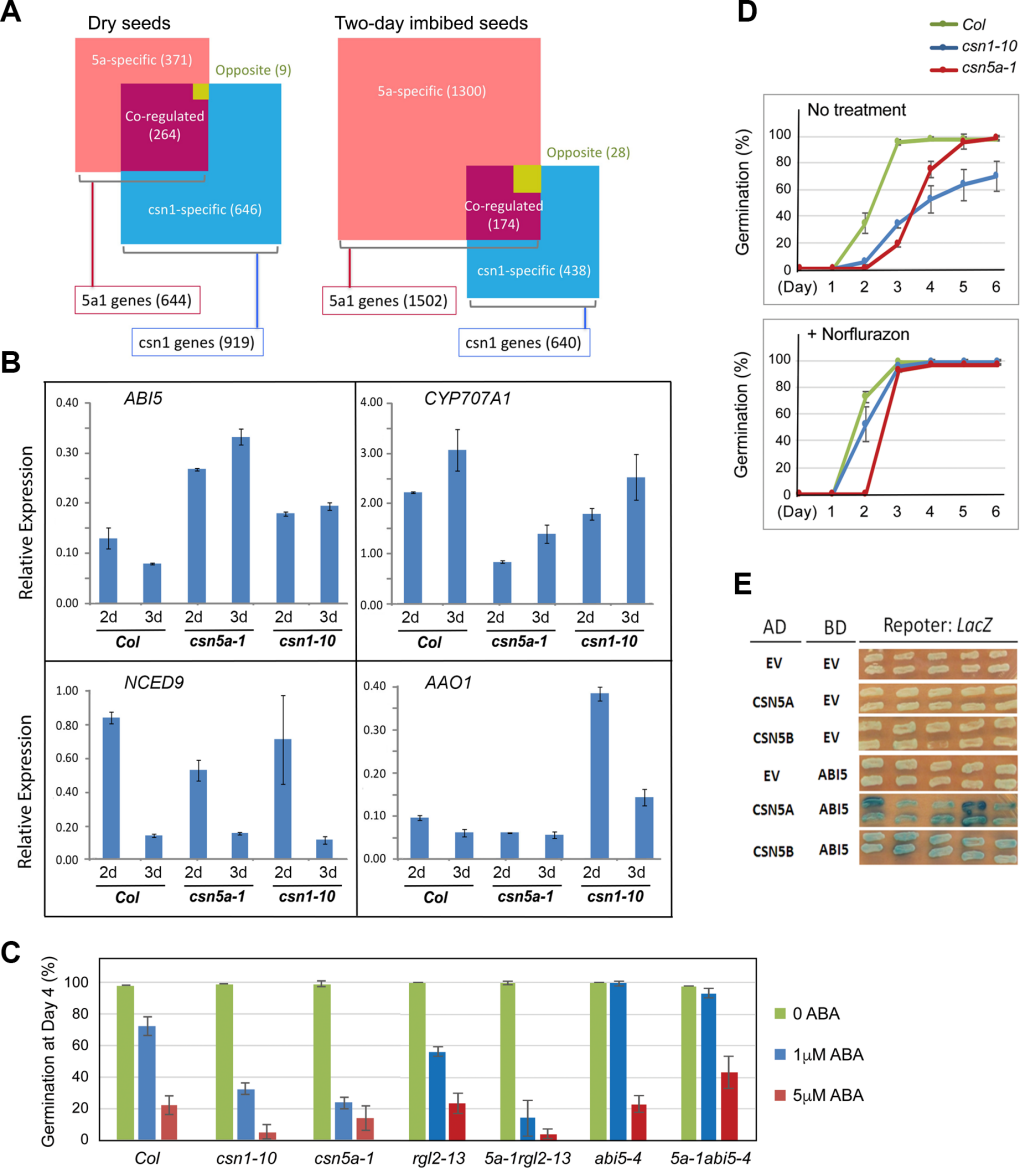
Effects of *csn1-10* and *csn5a-1* mutations on transcriptome and ABA sensitivity. **(A)** The diagrams showing the number of SSTF (statistically significant two-fold change) genes in *csn5a-1* and *csn1-10* mutants compared to wild type *Col* in dry seeds, or after two-day imbibition. *csn1-10* affected more genes than *csn5a-1* in dry seeds, while *csn5a-1* affected more genes than *csn1-10* in 2-day imbibed seeds. The areas of the squares were drawn in proportion to the number of the genes in the indicated category. **(B)** Real-time qPCR analysis of representative ABA related genes on day2 and day3 imbibed seeds of *Col*, *csn1-10*, and *csn5a-1*. The expression level of each sample was normalized to *IPA-like1* (AT1G17210). **(C)** Sensitivity to ABA-mediated inhibition of germination is enhanced in *csn1-10*, *csn5a-1*, and *csn5a-1rgl2* double mutant. The *csn5a-1abi5-4* double mutant showed resistance to ABA. Cold stratified seeds were used. Error bars represent standard deviation from 4 repeats. (**D)** ABA biosynthetic inhibitor norflurazon (5μM) rescued the low germination rates of both *csn1-10* and *csn5a-1*, but did not rescue the delayed germination in *csn5a-1*. Error bars represent standard deviation from 4 repeats. **(E)** Yeast-two-hybrid sassy showing interactions of ABI5 with CSN5A and CSN5B. AD, activation-domain fusion vectors; BD, DNA-binding domain fusion vectors; EV, empty vector. See also S6 and S7 Figs.

To understand the transcriptional changes in the *csn* mutants during germination, we conducted a transcriptome analysis on dry seeds and 2-day imbibed seeds of *csn5a-1* (or *5a-1*) and *csn1-10* along with the *Col* control. We determined the number of genes whose expression was significantly changed (SSTF, statistically significant two-fold change) in *csn1-10* or *csn5a-1* compared to the *Col* controls in dry seeds (**Fig 7A**) or in 2-day imbibed seeds (**Fig 7B**). *csn1-10* affected more genes (919 SSTF genes) than *csn5a-1* (644 SSTF genes) in dry seeds, suggesting that CSN1 plays a greater role than CSN5A in seed maturation. However, upon seed imbibition for two days, we observed a robust expansion in the number of genes whose expression was affected by the *csn5a-1* mutation (1502 SSTF genes) in comparison to those affected by *csn1-10 (*640 SSTF genes). This may indicate that CSN5A has more critical functions than CSN1 during germination and the active growth phase of Arabidopsis. This idea is also consistent with the expression value profiling analyses shown in heat-map plots (**S6 Fig)**. In dry seeds, *csn1-10* displayed greater abnormalities than *csn5a-1* in overall expression profiles when compared to that of *Col*, but in 2-day imbibed seeds, the profile of *csn5a-1* altered from that of *Col* more than *csn1-10* did. Also, *csn5a-1* exhibited an expression profile that appeared to have departed further from the dry seeds (**S6 Fig).** The top GO genes that are down-regulated in *Col* 2-day imbibed seeds compared to the dry seeds are genes responsive to various stimuli, and approximately the same GO catagory genes were further down-regulated in *csn5a-1* at the 2-day timepoint (**S1 Table**). *csn1-10*, on the other hand, did not show strongly enriched GO groups at the 2-day timepoint according to the *p*-values, but in dry seeds, it showed strong misregulation of temperature responsive genes (**S1 Table**).

### Functions of CSN and CSN5A in ABA regulation of germination

The transcriptome results showed that, from dry seeds to the 2-day time-point, several GA biosynthetic genes were strongly induced in *Col*, and ABA biosynthetic genes appeared to be active in dry and 2-day imbibed seeds (**S7 Fig**). However, we were unable to find drastic differences in the mutants. We also examined a panel of genes that have been shown to regulate dormancy and seed germination, but the *csn* mutants exhibited a similar pattern of expression changes as *Col* (**S7C Fig**). Since the seed germination conditions used for transcriptome analysis were not identical to that used for the germination tests, the kinetics of germination could differ slightly. We thus conducted qRT-PCR analysis of several ABA-related genes using the material from our standard germination testing conditions. At day2 and day3 post-imbibition, the ABA biosynthetic gene *AAO1* appeared to be expressed at higher levels in *csn1-10*, while the ABA catabolic gene *CYP707A1*, whose expression rose following seed imbibition, appeared to be lower in *csn5a-1* **(Fig 7B)**. The *ABI5* transcript was significantly down-regulated from dry seeds after 2days imbibition in *Col* as well as in both *csn* mutants (**S7C Fig**). On day2 and day3 post seed imbibition, the levels of *ABI5* were still moderately higher in both *csn* mutants compared to *Col* **(Fig 7B)**, although not as substantial as the difference in its protein accumulation.

We tested the sensitivity of the mutants to exogenous ABA during germination. On ABA-containing plates, both *csn5a1* and *csn1-10* were more sensitive to ABA than *Col* (**Fig 7C)**. In addition, the double mutant *csn5a-1 rgl2-13* was hyper-sensitive to ABA, whereas *csn5a-1 abi5-4* was hypo-sensitive to ABA. In a positive correlation, the mutants that were hypersensitive to ABA also showed abnormal accumulation of ABI5 (**Fig 4** and **Fig 5E**).

We were interested in the causes of the sustained ABI5 accumulation, a key inhibitor that has prevented timely germination of both *csn1-10 and csn5a-1* mutants. It has been shown that accumulation of RGL2 during germination can drive ABA biosynthesis, which would induce *ABI5* expression and inhibit germination [16]. To investigate whether de novo ABA synthesis is required for the germination phenotypes of the *csn* mutants, we applied ABA biosynthetic inhibitor norflurazon to test its effect on germination. As shown in **Fig 7D**, norflurazon treatment nearly completely restored the germination of *csn1-10*. While it considerably alleviated the germination defects of *csn5a-1*, norflurazon treatment could not rescue the delayed germination of *csn5a-1* **(Fig 7D)**. This result shows that *csn1-10*-induced hyperdormancy requires ABA synthesis after seed imbibition, which suggest that the extended ABI5 accumulation in *csn1-10* likely resulted from RGL2-induced ABA biosynthesis. By contrast, *csn5a-1* appeared to cause a defect downstream of ABA synthesis, which would support a role of CSN5A in facilitating ABI5 protein degradation following seed imbibition.

**Fig 8:**
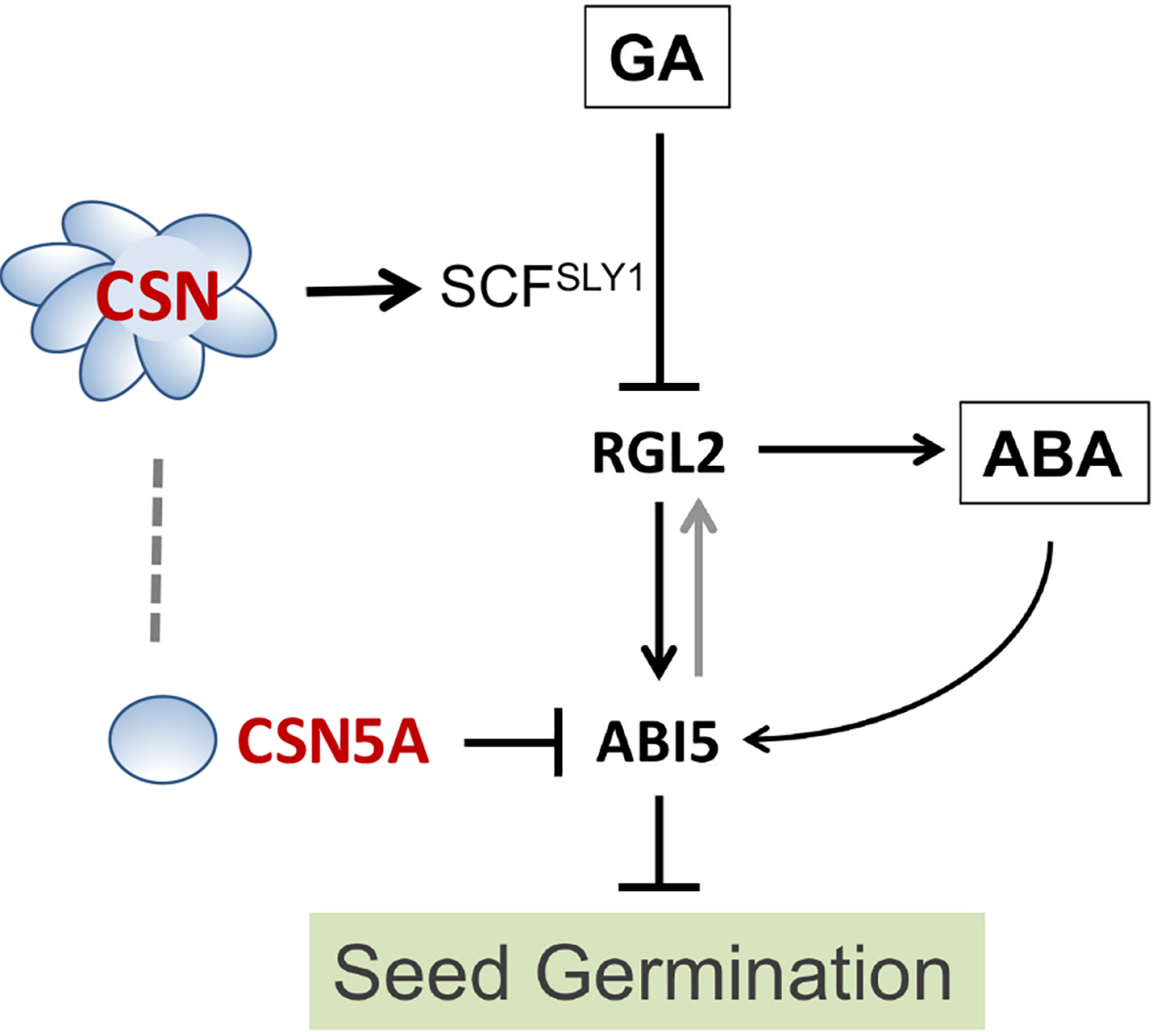
CSN functions in the seed germination pathway. A model indicating the targets of the CSN and CSN5A in the GA- and ABA- regulated seed germination pathway. The CSN complex (or CSN1) promotes germination by facilitating SCF-mediated degradation of RGL2 DELLA repressor. CSN5A, independently from the CSN complex, is involved in the turnover of ABI5, which acts downstream of RGL2. ABI5 appears to have a role in stabilizing RGL2, while RGL2 has been reported to activate ABI5 expression directly and by inducing ABA synthesis.

How CSN5A regulates ABI5 protein degradation is unclear. To this end, we found that CSN5A, or CSN5B, can interact with ABI5 in a yeast-two-hybrid assay (**Fig 7E**). The precise mechanism by which CSN5A regulates ABI5 will be a challenge for future studies. Taken together, we propose a model illustrating the hierarchical functions of the CSN in seed germination (**Fig 8)**. CSN1 and CSN5A, as parts of the CSN complex that regulates SCF ubiquitin E3s, are necessary for timely degradation of DELLA inhibitors, mainly RGL2. RGL2 inhibits germination by inducing ABA synthesis and promoting *ABI5* expression, based on previous reports. CSN5A additionally plays a role in the timely removal of ABI5 proteins to facilitate seed germination.

## Discussion

The COP9 signalosome is known to be necessary for efficient seed germination, but the molecular targets and the mechanism of its action are unclear [34, 39, 43]. We demonstrate here that the role of the CSN in promoting seed germination is by regulating two key inhibitors of seed germination, the DELLA protein RGL2 and the ABA effector ABI5. *csn1-10* and *csn5a-1* mutants are both poor in germination, but they exhibit subtly different germination phenotypes, and the mechanisms by which CSN1 and CSN5A regulate germination are not identical. *csn1-10* is hyper-dormant, while *csn5a-1* exhibits both hyperdormancy and delayed germination. CSN1 primarily regulates RGL2 turnover, which in turn affects ABI5 as a result. CSN5A is necessary in timely removal of ABI5 by a mechanism that apparently does not involve CSN1 (or the CSN complex). Thus, our study has identified the specific targets, from two different hormonal pathways, that underlie the germination defects of the *csn* mutants, and as such has clarified a long-standing mystery.

### CSN is required for timely degradation of RGL2 during seed germination

The CSN has been shown to regulate SCF family of ubiquitin E3 ligases. In fact, *csn1-10* and *csn3-3* were isolated as enhancers of *tir1-1*, a mutation of the auxin receptor that acts as the F-box component of SCF^TIR1^ [45, 46]. Thus, the CSN genetically promotes the function of SCF^TIR1^ in mediating auxin responses. Similarly, a *csn1* hypomorphic line showed genetic interactions with F-box proteins UFO and COI1 overexpression lines, supporting the role of the CSN in facilitating the functions of the corresponding SCF E3s [27, 40]. Moreover, *csn5a* and several lethal *csn* mutants were shown to abnormally accumulate RGA, a DELLA protein and a substrate of SCF^SLY1/2^ that has an important role in seedling development [43]. In the same report, the germination defect of the *csn* mutants were described, but the molecular targets responsible for their germination phenotypes were not determined. In this study, we show that the inability to timely degrade RGL2, a key inhibitor of germination for dormancy response and a substrate of SCF^SLY1/2^, can fully account for the hyper-dormant phenotype of *csn1-10*. Considering what is known about the function of the CSN, we suggest that CSN1, or the CSN complex, most likely plays an important role in SCF^SLY1/2^-mediated RGL2 ubiquitination during the course of seed germination (**Fig 8**).

### CSN5A regulates ABI5 downstream of CSN-facilitated degradation of RGL2 during germination

In Arabidopsis, complete loss of any one of the eight canonical subunits of the CSN leads to the destruction of the complex [32]. This explains why null mutants of any one of the CSN subunits all cause early seedling lethality, with a similar “fusca” phenotype. Probably as a consequence of this similarity, all CSN subunits have largely been indiscriminately viewed to have more or less same physiological functions in plants.

In a number of other species, differences in phenotypes and functions for different subunits of the CSN have been observed [53]. In fungi, deletion mutants of different CSN subunits cause distinct phenotypes [54, 55]. In mammalian cells, knock-down of CSN3 or CSN8 in cultured cells can accelerate cell proliferation [56, 57], whereas knock-down of CSN5 decreases cell proliferation and causes cell senescence [57, 58]. The human CSN5 has been extensively studied due to its association with many types of cancers [59]. Originally isolated in human cells as a coactivator of c-Jun, Jab1 (c-Jun activation domain-binding protein 1) [60], CSN5/JAB1 has been reported to bind to an array of transcription factors or other critical cellular regulators to stabilize the target protein or to facilitate its degradation. As an integral subunit of the CSN complex, CSN5 carries the catalytic center of the CSN deneddylase that is active only when assembled into the complex [61]. It remains unclear how the regulatory activities of CSN5 on its binding partners are related to the role of CSN5 in deneddylation of the CRL E3 ligases. Notably, in a highly conserved manner from yeast to plants and mammals, CSN5 has been found to exist both as part of the holo-CSN complex and in a free form unbound to the CSN [53]. Studies in mammalian systems have shown that CSN5 has functions independent of the CSN holocomplex [58]. Whether the Arabidopsis CSN5, like its mammalian counterpart, has functions apart from that of the CSN complex, has not been reported.

In Arabidopsis, *csn1-10* and *csn5a-1* both exhibit poor germination. However, careful analyses indicate that the two mutants differ in several aspects. First, *csn1-10* shows deeper seed dormancy, such that its germination can be restored by cold stratification or extended after-ripening period, whereas *csn5a-1* additionally exhibits a delayed germination that is resistant to these dormancy-breaking measures **(Fig 2 and S2 Fig)**. Second, the *csn1-10* germination phenotype can be suppressed by loss of RGL2, while *csn5a-1* cannot (**Fig 5A and 5B**). Third, the responses to GA-synthesis inhibitor PAC and to ABA-synthesis inhibitor norflurazon are different. In particular, norflurazon completely rescues the germination of *csn1-10*, but cannot rescue the delayed germination of *csn5a-1* (**Fig 7D**). We suggest that the different behaviors between the two mutants can be explained by *csn5a-1*-specific deficiency in protein degradation of ABI5, which occurs downstream of RGL2 in the germination pathway **(Fig 8)**. PAC treatment has been shown to stabilize RGL2 and stimulate accumulation of ABI5 [16]. That *csn5a-1* mutants failed to down-regulate ABI5 protein would render the mutant hypersensitive to PAC. As a consequence, even though *rgl2* can confer resistance to PAC in wild type background, *csn5a-1 rgl2* double mutant still accumulate ABI5 and consequently remain hypersensitive to PAC. The fact that *rgl2* can fully rescue the germination of *csn1-10 rgl2* suggests that CSN1 does not play a significant role in direct regulation of ABI5. The prolonged ABI5 accumulation in *csn1-10* mutant is most likely caused by its extended accumulation of RGL2, which induce ABA biosynthesis and promote *ABI5* expression.

The fourth difference between the *csn1-10* and *csn5a-1* is implied by their transcriptome profiles, which indicate that the two mutations have differential impacts on plants at different developmental stages. Relative to each other, *csn1-10* strongly affected seed maturation, while *csn5a-1* strongly affected seed germination and seedling establishment (**Fig 7A**).

We also found a peculiar behavior of *csn5b-1*, which has a weaker seed dormancy than *Col* (**Fig 2**), opposite to the phenotype of the rest of the *csn* mutants. Similar phenomena have been reported regarding the root phenotype. It was observed that *csn5b-1* has more adventitious roots, opposite to that of other *csn* mutants which develop fewer (*csn1-10* and *csn3-3*) or no (*csn5a-1* and *csn5a-2*) adventitious roots [42]. It is possible that CSN5B has a different function from CSN5A, which results in the difference of the phenotype. Alternatively, it is possible that *csn5b-1* only slightly reduces the activity of CSN to the extent that could not be definitively detected by immunoblotting. Since CSN’s de-neddylation activity biochemically inhibits SCF, the slight reduction of the CSN might increase the SCF activity without creating a hyperactive SCF that auto-ubiquitinates its own components, as in severe *csn* mutants. This might enhance the SCF activity in targeting RGL2, resulting in the reduced dormancy in *csn5b-1*. This idea is admittedly highly speculative.

### CSN5 has unique functions in ABA signaling

With this study, we have for the first time revealed that CSN5A can directly or indirectly regulate protein stability of the b-ZIP transcription factor ABI5 (**Fig 8**), a key transcription factor that mediate the response to ABA, especially in seed germination [20, 62]. Elevated ABI5 may attribute to the hypersensitivity of the *csn1-10*, *csn5a-1* and *csn5a rgl2* in ABA-mediated inhibition of germination **(Fig 7C).** It has been reported that ABA receptor is targeted for degradation by the CRL4-CDDD E3 complex [63]. Given that CSN can regulate CRL4, it seems possible that CRL4’s activity in targeting the ABA receptor be compromised in the *csn* mutants, which might also contribute to the ABA hypersensitivity.

*ABI5* transcripts are drastically down-regulated following seed imbibition in the *csn* mutants (**S7C Fig**), although the levels are still moderately higher than in the wild type at day2 and day3 after imbibition (**Fig 7B**). Nonetheless, the elevated level of *ABI5* transcripts cannot fully account for the sustained high-level accumulation of its protein in the mutants (**Fig 4**). Moreover, inhibiting ABA biosynthesis, and thus ABA-induced *ABI5* expression, cannot rescue the delayed germination of *csn5a-1*. These results suggest that the sustained ABI5 accumulation is probably caused by defective ABI5 degradation in *csn5a-1*, which results in a delay of the germination.

ABI5 protein stability has been shown to be regulated by several factors and ubiquitin E3 ligases, including *KEEP ON GOING* (*KEG*) [64], *SALT- AND DROUGHT-INDUCIBLE RING FINGER 1* (*SDIR1*) [65], *ABI FIVE BINDING PROTEIN* (*AFP*) [66], CRL4^ABD^ and CRL4^DWA1/2^ [67] [68]. In these studies, KEG and CRL4s ubiquitin E3 ligases are shown to target ABI5 in post-germination events. Our data suggest that CSN5A facilitates protein degradation of ABI5 during germination, but it is unclear which of those known ABI5 E3 ligases CSN5A works with. We also cannot exclude that there may be a different E3 yet to be identified that targets ABI5 specifically during germination. Furthermore, ABI5 protein undergoes various post-translational modifications including phosphorylation, SUMOylation and S-nitrosylation, all of which can modulate its protein stability [62, 69, 70]. It cannot be ruled out that CSN5A may modulate ABI5 protein stability by affecting those modifications on ABI5.

Our findings raise additional questions as to whether CSN5A may affect other aspects of ABI5 functions or other ABA responses. Regardless, the observations that ABI5 is specifically affected in *csn5a* but not in *csn1* suggest that this function might represent the first CSN5-specific activity in plants. With this function of CSN5A, we can now add ABA responses to the repertoire of CSN/CSN5 regulated pathways.

## Materials and methods

### Plant material and growth conditions

The Arabidopsis thaliana mutants *csn5a-1*, *csn5a-2*, *csn5b-1* [35], *csn1-10* [45], *csn3-3* [46], and *rgl2-13* [13] are in the *Col* ecotype. *csn2-5* [47] is in Langsberg *eracte* (*Ler*) background. The *abi5-4* and *csn1-1* (*fus6-1*) mutants are in Wassilewskija (*Ws*) ecotype. After the seeds were germinated and grown on GM plates (see below) for 6 to 9 days, the seedlings were transferred to soiled pots. The soil mix was composed of one part Vermiculite and one part Farfard#2 soil mix, which were soak in water with fertilizer (0.25 g/L water). The fertilizer is from Scotts (Peters Professional 20-10-20 #99250 from Scotts). Plants were grown in a walk-in growth room in 22°C long day light cycle.

### Germination assays

When not specified, seeds between 1 week to 6 weeks were used and un-stratified in the germination tests or immunoblot analyses. For experiments requiring cold stratification to break the dormancy, such as testing sensitivity to PAC or ABA, seeds from 1 week to up to 6 month were used, and the seeds were cold stratified for 2–3 days regardless of the collecting time. Seeds to be used for direct comparisons were usually collected on the same day from the parental plants grown side-by-side under the same conditions. This was strictly the case for *Col* and *csn1-10*. However, since *csn5a-1* plants is late–flowering, it was matched with later planted *Col* that had a same or close seed collection dates. Seeds were surface sterilized with a solution containing 30% bleach and 0.1% TritonX-100, washed. When not specified, seeds are saw on solid growth medium (GM) containing MS salt 4.4 g/L (M0404, Sigma), MES 0.5g/L (M5287, Sigma), 1% sucrose and 0.8% (w/v) Bacto-Agar. The plates were divided to 4 areas to aid in counting and scoring seed germination rates. To determine the germination rates, approximately 20–60 seeds in 4 repeats were examined daily under a Nikon/Zeiss dissecting microscope, and germination were scored based on radicle protrusion from endosperm. The germination rates were measured in DAI (days after imbibition). Seeds to be tested for germination were routinely carried out in pairs: one set of the seeds were un-stratified and the germination rates were presented unless otherwise noted; the other matching set of seeds were cold stratified (4°C) in the dark for 3–4 days, and their germination rates were recorded to make sure that the batch of the seeds were of high quality. Plates were incubated in a Percival growth chamber for 22°C with constant white light.

For PAC, GA, and ABA sensitivity test, cold stratified seeds were used. The medium was supplemented with PAC (paclobutrazol, sc-236284, Santa Cruz Biotechnology), GA3 (G7645 Sigma). For ABA sensitivity teste, solid GM medium described above without sucrose were used that contained ABA (A1049, Sigma-Aldrich) in indicated concentration. For testing of norflurazon (Sigma-Aldrich 34364) effect, unstratified seeds were used on no sucrose GM plates.

For light controlled seed germination experiment, seeds sow on GM plates were cold stratified for two days in the dark, then seeds were exposed to 5 min red light (13.4 watts.m^-2^) or far-red light (8.2 watts.m^-2^), or followed by another 5 min of light treatment as indicated. Plates containing the seeds were then wrapped in foil and kept in the dark at 22°C incubator for two days before counting for germination.

### Seed coat dissection experiments

After sterilizing and washing, seeds were imbibed in water for 3 hours before dissection using a fine syringe needle according to a previously described procedure [51, 52]. Dissected embryos were placed on a water agar (1%) plate alone with corresponding seed controls, and were incubated in a 22°C growth chamber with constant white light. Embryos and seeds were examined through a Leica dissecting microscope and photographs were taken daily.

### Protein extraction, antibodies and immunoblot analysis

For every 100 mg of fresh weight imbibed seeds or germinating seedlings, the sample was mixed with 180 microliter of chilled extraction buffer (50mM Tris-HCL, pH7.5, 150mM NaCl, 10mM MgCl2, 2.5mM EDTA, 1mM DTT, 0.1% Nonidet P-40, and freshly added 1mM protease inhibitors phenylmethylsulfonyl fluoride (PMSF) and 1x complete protease inhibitor cocktail (Roche Molecular Biochemicals) was added and mixture was homogenized. Then, 100 microliter of 4x sample buffer was added and the mixture was vortexed. Samples were then boiled for 10 min and span in a microfuge for 10 min. The supernatant was transferred to a new tube, from which samples were loaded onto SDS-PAGE for immunoblotting.

Antibodies used for this study include anti-CSN5A and anti-CSN3 [35], anti-CUL1 [48], anti-RPN6 [71], anti-RGL2 [72], and anti-ABI5 (Abcam, ab98831). The anti-CSN5B polyclonal antibodies were made by Beijing Protein Innovation Co., Ltd. (BPI). Briefly, an EcoRI/XhoI fragment containing the full-length CSN5B open reading frame was cloned into the EcoRI/XhoI sites of the pET-28a vector, so as to express 6×His-tagged CSN5B protein. The fusion protein was expressed in *Escherichia coli*, then purified and used as antigen to immunize rabbits for the production of polyclonal antiserum. Antigen affinity purified anti- CSN5B antibodies were used in immunoblots.

### Transcriptome analyses by RNA-seq

*Col-0*, *csn1-10*, and *csn5a-1* seeds were collected from plants grown side-by-side in the growth room. Seeds for *Col-0* and *csn1-10* were 1.5wks in storage, and *csn5a-1* seeds were 4wk in storage. Approximately 100 microliter of settled seeds were used for RNA extraction for each sample. The 2-day imbibed seeds, prepared with the same volume of dry seeds as above, were sterilized, washed, and incubate on cell culture wells with 1ml of 0.5X liquid MS medium at 22°C under constant light for 2 days. Each sample points had three biological repeats. Seeds were centrifuged to remove the liquid, and were frozen in liquid nitrogen. The frozen seeds were ground to a fine powder using mortar and pestle in the presence of liquid nitrogen and small quantity of sterile quartz powder. RNA extraction was performed according to a published procedure [73].

High-throughput RNA-seq was carried out at Yale Center for Genome Analysis. The Single-End RNA-sequencing was carried out with Illumina Hi-seq 2000 platform (Genome Center, Yale West Campus). Specifically, libraries were analyzed with a Bioanalyzer 2100 instrument (Agilent, Santa Clara, CA), quantified by Qubit fluorometer (Life Technologies, Carlsbad, CA). The *Arabidopsis thaliana* genome obtained from TAIR10 (https://www.arabidopsis.org) was used as the genome reference. After adaptor trimming and contaminate sequence removing by fastqc (www.bioinformatics.babraham.ac.uk/projects/fastqc/) and fastx-toolkits (hannonlab.cshl.edu/fastx_toolkit/), Bowtie2 (http://bowtie-bio.sourceforge.net/bowtie2/index.shtml) was used for genome mapping and followed by the tophat (https://ccb.jhu.edu/software/tophat/index.shtml) transcript assembling. Gene differentially expression profiling was accomplished by cufflink and cuffdiff software package (cole-trapnell-lab.github.io/cufflinks/cuffdiff/) with default parameters and cutoffs, fold change cutoff was set to 2. The data set is accessible at NCBI GEO under accession number GSE106223.

### qRT-PCR

*Col*, *csn5a-1* and *csn1-10* seeds (5-days in storage) were sterilized and sow on the solid medium plate as described above in Germination assay. Total RNAs were extracted from 2- or 3- day germinating *Col*, *csn5a-1* and *csn1-10* seeds following the procedures as described previously [73]. Then 1 μg of total RNA was used for reverse transcription reaction using SuperScript III Reverse Transcriptase (Invitrogen) and quantitative PCR (qPCR) reaction was performed in Bio-Rad CFX96 real-time system using iQ SYBR Green mix (Bio-Rad). Gene expression was normalized to *IPA-like1* (AT1G17210).

### Yeast-two-hybrid assay

Full-length cDNA coding region of *CSN5A* and *CSN5B* were each subcloned into the *Eco*RI/*Sal*I and *Eco*RI/*Xho*I sites of pB42AD(AD) vector (Clontech). *ABI5* was subcloned into the *Eco*RI/*Xho*I site of pLexA (BD) vector (Clontech). Yeast two-hybrid assay was performed according to the Matchmaker LexA Two-Hybrid System manual (Clontech, K1609-1). Briefly, all constructs were co-transformed into yeast strain EGY48 containing p8op-LacZ. Transformants were grown on SD/-His/-Trp/-Ura plates containing X-Gal for blue color development.

## Supporting information

S1 Table Top enriched GO terms in imbibed seeds of *csn5a-1* and *csn1-10* mutants, in comparison to *Col*. (DOCX)

S2 Table Accession numbers of the genes mentioned in the study. (DOCX)

S3 Table Primer sequences used for genotyping and RT-qPCR. (DOCX)

S1 Fig Adult plant phenotype of viable *csn* mutants and expression level of the CSN in various plant tissues. **(A)** Adult phenotypes of 5-weeks *csn* mutant plants, compared to the wild type plants of their corresponding ecotype backgrounds. *csn5a-1*, *csn5a-2*, *csn5b-1*, *csn1-10*, *csn3-3* are in *Col* background, while *csn2-5* is in *Ler* background. **(B)** Expression of CSN subunits in various tissues of Arabidopsis in wild type and *csn5* mutants. Tissues were collected from wild type (Col-0) or *csn5a2 or csn5b1* mutants as indicated. Total proteins were analyzed by immunoblotting using antibodies against CSN5A, CSN1, and CSN8. Anti-RPN6 blots on the same samples were used as an internal reference. *csn5b-1* mutation had no detectable effect on the level of CSN subunits in the tissues examined. *csn5a-2* mutation resulted in notable reduction in CSN5A level, and had minor effect on CSN1 and CSN8 levels.
(JPG)

S2 Fig Cold stratification suppressed the hyperdormant germination of *csn1-10* and *csn5a-1* regardless of the sucrose content in the medium, but cannot suppress the retarded seed germination of *csn5a-1*. **(A)** *Col*, *csn1-10* and *csn5a-1* seeds (4-day after collection) were tested for germination on solid growth medium containing 1% sucrose (left panels), or had no sucrose (right two panels). Seeds were not stratified (top panels) or cold stratified for 4 days (bottom panels). The slow germination phenotype was clearly displayed on both sucrose-containing or sucrose-less plates. **(B)** *Col* and mutant seeds of indicated storage age were cold stratified for 4 days before the germination test. *csn5a-1* showed delayed germination even after cold stratification of fully after-ripened seeds.
(JPG)

S3 Fig Phytochrome-B mediated control of seed germination is normal in the *csn* mutants tested. **(A)** The diagram illustrating the light treatment procedure used to test phyB-controlled seed germination. Seeds were cold stratified for 2 days prior to the light treatment. After light treatments, seeds were incubated in the dark at 22oC for two or three days. WL, constant white light; R, red light 5 min; FR, far-red light 5min; FR-R, far-red light 5min followed by red light 5 min; FR-R-FR, far-red light 5min followed by red light 5 min and followed by far-red light 5 min. **(B)** The germination rates at day-2 (or day-3 for *csn5a-1*) post light treatments are shown. The color code in **(B)** is identical to the colors indicating light treatment in **(A)**. The *csn* mutants exhibited largely normal light responsive seed germination, while *det1-1* (as a control) showed light-independent seed germination. Error bars represent standard deviation from 4 repeats.
(JPG)

S4 Fig *rgl2-13* can significantly rescue the germination defect of *csn1-1*, a null mutant of *csn1*. The graph shows the germination rates at day-10 and day-12 of two segregating sibling lines of *csn1-1* and double mutant *csn1-1 rgl2-13*. Error bars represent standard deviation from 4 repeats.
(JPG)

S5 Fig *abi5-1* can rescue the germination delay of *csn5a-2*. In the right panel, the *csn5a-2 abi5-1* double mutants showed improved germination rates over *csn5a-2*. These mutant lines were segregating sibling lines from the same cross. Germination rate of one representative *csn5a-2abi5-1* double and *csn5a-2* single mutant lines were counted and graphed as shown on the left. Error bars represent standard deviation from 4 repeats.
(JPG)

S6 Fig The transcriptome profile of *csn1-10* and *csn5a-1* in dry seeds and 2-day imbibed seeds. **(A)** Heatmap diagram of mRNA profiles in dry seeds of Col wild type, *csn1-10*, and *csn5a-1*. Genes with expression values from 13 and above were included. The color code is set from 13–300 in Log2 scale. Genes with expression level above 300 show the same color as those of 300 (red). The transcriptome profile of *csn1-10* in dry seeds show greater dissimilarity to wild type than comparing *csn5a-1* to wild type. **(B)** Heatmap plot showing gene expression profile in 2-day imbibed seeds of *csn5a-1*, *csn1-10*, and *Col*, compared to *Col* dry seeds. Genes with the expression value from 13 and above were included. The color code is set from 13 to 300 in Log2 scale. Genes with expression values above 300 are indicated with the same color as those of 300 (red). *csn5a-1* displayed greater transcriptome changes than *csn1-10* did at 2-day post imbibition.
(JPG)

S7 Fig Expression of ABA, GA- related genes or known seed germination regulators in *csn5a-1* and *csn1-10* mutants from transcriptome datasets. **(A and B)** Heatmap showing ABA-related genes **(A)** and GA related genes **(B)** in 2-day imbibed seeds of *csn5a-1 (5a-1)*, *csn1-10*, and *Col*, compared to those of *Col* dry seeds. **(C)** A panel of known seed germination regulatory genes showed similar expression profiles in *csn5a-1*, *csn1-10* and *Col*. These genes are: *DAG1 (DOF AFFECTING GERMINATION 1 AT3G61850), DOG1 (DELAY OF GERMINATION 1, AT5G45830), SPY SPINDLY, AT3G11540), SPT SPATULA, AT4G36930), LDL1/2 (ARABIDOPSIS LYSINE-SPECIFIC HISTONE DEMETHYLAS AT1G62830/ AT3G13682), MFT (MOTHER OF FT AND TFL, AT1G18100), RGL2 (RGA-LIKE 2, AT3G03450), RDO5 (REDUCED DORMANCY 5, AT4G11040), ABI5 ABA INSENSITIVE 5, AT2G36270)*.
(JPG)
