# Supplementary material for: The COP9 Signalosome regulates seed germination by facilitating protein degradation of RGL2 and ABI5
Source: PLoS Genet. 2018 Feb 20;14(2):e1007237. doi: 10.1371/journal.pgen.1007237 (PMC5834205; doi:10.1371/journal.pgen.1007237)
Supplement: S1 Table — (DOCX) [file pgen.1007237.s001.docx]

| **Table 1. Top 5 GO terms in *csn* mutant seeds** | | |  |  |  |
| --- | --- | --- | --- | --- | --- |
| p-value color key | | 9E-20 to 1E-50 |  |  |  |
|  |  | 9E-11 to 1E-19 |  |  |  |
|  |  | 9E-08 to 1E-10 |  |  |  |
|  |  | 9E-04 to 1E-07 |  |  |  |
|  |  | 9E-01 to 1E-03 |  |  |  |
|  |  |  |  |  |  |
|  |  | Top GO terms | p-value | No. genes | GO ID |
| **WT** | Induced | biosynthetic process | 1.89E-48 | 474 | 9058 |
| ***(Col)*** | 2-day/dry | cellular biosynthetic process | 6.05E-48 | 451 | 44249 |
|  |  | gene expression | 9.64E-48 | 335 | 10467 |
|  |  | translation | 4.79E-42 | 256 | 6412 |
|  |  | cellular metabolic process | 8.80E-42 | 774 | 44237 |
|  | Repressed | response to chemical stimulus | 6.25E-10 | 281 | 42221 |
|  | 2-day/dry | response to heat | 6.47E-10 | 45 | 9408 |
|  |  | response to abiotic stimulus | 1.06E-09 | 205 | 9628 |
|  |  | response to stimulus | 1.11E-09 | 467 | 50896 |
|  |  | response to temperature stimulus | 7.20E-09 | 83 | 9266 |
| **Imbibed** | Induced | generation of precursor metabolites and energy | 2.45E-07 | 16 | 6091 |
| **2-day** | *5a/Col* | cellular nitrogen compound metabolic process | 4.40E-07 | 46 | 34641 |
|  |  | nitrogen compound metabolic process | 5.26E-07 | 47 | 6807 |
|  |  | response to red light | 1.54E-05 | 8 | 10114 |
|  |  | respiratory electron transport chain | 2.81E-05 | 6 | 22904 |
|  | Repressed | response to temperature stimulus | 4.11E-15 | 43 | 9266 |
|  | *5a/Col* | response to heat | 4.22E-13 | 25 | 9408 |
|  |  | response to high light intensity | 4.53E-13 | 16 | 9644 |
|  |  | response to chemical stimulus | 2.14E-11 | 93 | 42221 |
|  |  | response to light intensity | 2.14E-11 | 18 | 9642 |
|  | Induced | cellular component biogenesis | 2.06E-02 | 11 | 44085 |
|  | *csn1/Col* | DNA methylation on cytosine | 3.38E-02 | 2 | 32776 |
|  |  | RNA metabolic process | 4.46E-02 | 11 | 16070 |
|  |  |  |  |  |  |
|  | Repressed | response to stress | 1.54E-02 | 26 | 6950 |
|  | *csn1/Col* | response to abiotic stimulus | 1.54E-02 | 19 | 9628 |
|  |  | hyperosmotic response | 2.36E-02 | 4 | 6972 |
|  |  | cell wall macromolecule catabolic process | 2.36E-02 | 3 | 16998 |
|  |  | response to temperature stimulus | 2.36E-02 | 9 | 9266 |
| **Dry** | Induced | cellular component biogenesis | 2.03E-05 | 14 | 44085 |
| **Seeds** | *5a/Col* | response to stimulus | 9.29E-05 | 39 | 50896 |
|  |  | ncRNA metabolic process | 9.29E-05 | 9 | 34660 |
|  |  | response to light stimulus | 9.29E-05 | 13 | 9416 |
|  |  | response to abiotic stimulus | 1.11E-04 | 21 | 9628 |
|  | Repressed | response to heat | 1.82E-05 | 10 | 9408 |
|  | *5a/Col* | response to temperature stimulus | 1.31E-02 | 11 | 9266 |
|  |  | response to chitin | 1.31E-02 | 6 | 10200 |
|  |  | response to endogenous stimulus | 1.31E-02 | 17 | 9719 |
|  |  | response to hormone stimulus | 1.31E-02 | 16 | 9725 |
|  | Induced | cytokinesis | 2.60E-03 | 5 | 910 |
|  | *csn1/Col* | response to abiotic stimulus | 3.16E-03 | 26 | 9628 |
|  |  | microtubule-based process | 2.03E-02 | 6 | 7017 |
|  |  | RNA metabolic process | 2.03E-02 | 14 | 16070 |
|  |  | response to UV-B | 2.50E-02 | 4 | 10224 |
|  | Repressed | response to heat | 1.79E-11 | 16 | 9408 |
|  | *csn1/Col* | response to temperature stimulus | 1.47E-08 | 20 | 9266 |
|  |  | response to hydrogen peroxide | 1.02E-05 | 7 | 42542 |
|  |  | response to high light intensity | 1.08E-05 | 7 | 9644 |
|  |  | response to light intensity | 2.70E-05 | 8 | 9642 |
