# Supplementary material for: The COP9 Signalosome regulates seed germination by facilitating protein degradation of RGL2 and ABI5
Source: PLoS Genet. 2018 Feb 20;14(2):e1007237. doi: 10.1371/journal.pgen.1007237 (PMC5834205; doi:10.1371/journal.pgen.1007237)
Supplement: S2 Table — (DOCX) [file pgen.1007237.s002.docx]

**Table S2 Accession numbers of the genes mentioned in the study**

| **Genes** | **Accession numbers** | |  | **Genes in S6 Figure** | **Accession numbers** |
| --- | --- | --- | --- | --- | --- |
|  |  | |  |  |  |
| *CSN1* | AT3G61140 | |  | *AO4* | AT1G04580 |
| *CSN5a* | AT1G22920 | |  | *ABA1* | AT5G67030 |
| *CSN5b* | AT1G71230 | |  | *XERICO* | AT2G04240 |
| *CSN2* | AT2G26990 | |  | *AAO3* | AT2G27150 |
| *CSN3* | AT5G14250 | |  | *ABA4* | AT1G67080 |
| *CSN8* | AT4G14110 | |  | *SDR3* | AT2G47130 |
| *CUL1* | AT4G02570 | |  | *ABA3* | AT1G16540 |
| *RPN6* | AT1G29150 | |  | *AAO2* | AT3G43600 |
| *IAP-like1* | AT1G17210 | |  | *AO1* | AT5G20960 |
| *RGL2* | [AT3G03450](http://www.arabidopsis.org/servlets/TairObject?id=40021&type=locus) | |  | *NCED2* | AT4G18350 |
| *ABI5* | [AT2G36270](http://www.arabidopsis.org/servlets/TairObject?id=32860&type=locus) | |  | *NCED3* | AT3G14440 |
| *DAG1* | [AT3G61850](http://www.arabidopsis.org/servlets/TairObject?id=36500&type=locus) | |  | *NCED6* | AT3G24220 |
| [*DOG1*](http://www.arabidopsis.org/servlets/TairObject?id=131700&type=locus) | AT5G45830 | |  | *NCED9* | AT1G78390 |
| *SPY* | [AT3G11540](http://www.arabidopsis.org/servlets/TairObject?id=36698&type=locus) | |  | *CYP707A1* | AT4G19230 |
| *LDL1* | *AT1G62830* | |  | *CYP707A2* | AT2G29090 |
| *LDL2* | AT3G13682 | |  | *ABA2* | AT1G52340 |
| *MFT* | [AT1G18100](http://www.arabidopsis.org/servlets/TairObject?id=136229&type=locus) | |  | *PYL8* | AT5G53160 |
| *RDO5* | [AT4G11040](http://www.arabidopsis.org/servlets/TairObject?id=129637&type=locus) | |  | *CYP88A3* | AT1G05160 |
| *CYP707A1* | AT4G19230 | |  | *GA3OX1* | AT1G15550 |
| *NCED9* | AT1G78390 | |  | *GA2OX2* | AT1G30040 |
|  |  | |  | *GA2* | AT1G79460 |
|  |  | |  | *GA3OX2* | AT1G80340 |
|  |  | |  | *PIL5* | AT2G20180 |
|  |  | |  | *KAO2* | AT2G32440 |
|  |  | |  | *ATGA2OX3* | AT2G34555 |
|  |  | |  | *GA2OX8* | AT4G21200 |
|  |  | |  | *GA20OX1* | AT4G25420 |
|  |  | |  | *CTR1* | AT5G03730 |
|  |  | |  | *GA20OX3* | AT5G07200 |
|  |  | |  | *GA3* | AT5G25900 |
|  |  | |  | *AT5G51310* | AT5G51310 |
|  |  | |  | *GA20OX2* | AT5G51810 |
|  |  | |  | *Tudor2* | AT5G61780 |
|  |  | |  | *ABC33* | AT4G02780 |
|  |  | |  |  |  |
|  | |  |  |  |  |
