# Supplementary material for: The COP9 Signalosome regulates seed germination by facilitating protein degradation of RGL2 and ABI5
Source: PLoS Genet. 2018 Feb 20;14(2):e1007237. doi: 10.1371/journal.pgen.1007237 (PMC5834205; doi:10.1371/journal.pgen.1007237)
Supplement: S3 Table — (DOCX) [file pgen.1007237.s003.docx]

Table S1. Genotyping and RT-qPCR primers

| **Mutants** | **alleles** | **primers** | **sequence** |
| --- | --- | --- | --- |
| *csn5a-1* | wt | A5 | CTCTTCTTTTACGCTTTGTGATTT |
|  |  | A6 | CATAACCAATCAATCAAACACTCG |
|  | mut | A5 | CTCTTCTTTTACGCTTTGTGATTT |
|  |  | LBb1 | GCGTGGACCGCTTGCTGCAACT |
| *csn5a-2* | wt | 5a2F | GGCAATGGAGACTATGTTGC |
|  |  | 5aYR | TCACGATGTAATCATGGGC |
|  | mut | 5a2F | GGCAATGGAGACTATGTTGC |
|  |  | LBb1 | GCGTGGACCGCTTGCTGCAACT |
| *csn5b-1* | wt | B1 | CCCGAAGTAACATTTTTGACACAG |
|  |  | B2 | TTAGGCCATATCCAAGAGTTTACC |
|  | mut | B2 | TTAGGCCATATCCAAGAGTTTACC |
|  |  | LBb1 | GCGTGGACCGCTTGCTGCAACT |
| *rgl2-13* | wt | Nt250 | GTTCTACTGTCTTAAACGACTCTGTTCATT |
|  |  | RGL2 C-R | ATCGGTACCGCCGCGACTCAGG |
|  | mut | Nt250 | GTTCTACTGTCTTAAACGACTCTGTTCATT |
|  |  | LBb1 | GCGTGGACCGCTTGCTGCAACT |
| *abi5-4* | Wt/mut | ABI5nt76 | GGAGGTGGTGGTGAGAATCA |
|  |  | ABI5nt460 | TACAAAGCGGAGCTGGAAGT |
| **Genes** | **Primer sequences** | | |
| IPA-like1 | TGTGTCGTTGACGAAGGAGG and ATCTAGCACGTCGTCGGAAC | | |
| NCED9 | GGTACCGGAGAAACCTGTCG and CGTGTACCATACCGTCTCCG | | |
| CYP707A1 | TGGCCTTACGTCGGAGAAAC and CTTAGCAGCCTCTGGACTCG | | |
| ABI5 | CAGCTGCAGGTTCACATTCT and TCGTTCGCTATCCCTCTCTT | | |
