# Supplementary figures and images for: The COP9 Signalosome regulates seed germination by facilitating protein degradation of RGL2 and ABI5
Source: PLoS Genet. 2018 Feb 20;14(2):e1007237. doi: 10.1371/journal.pgen.1007237 (PMC5834205; doi:10.1371/journal.pgen.1007237)

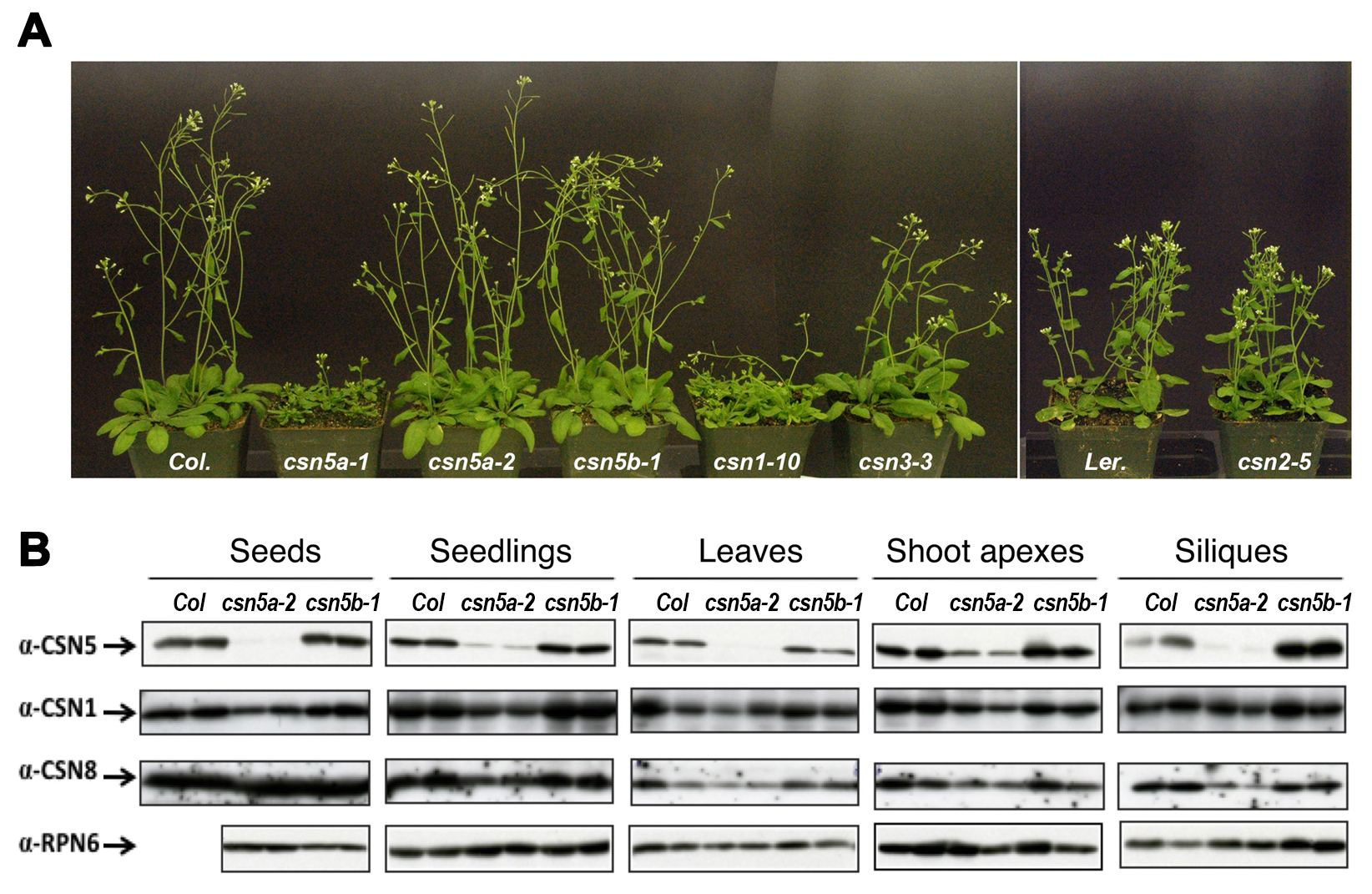

Supplement: S1 Fig — (A) Adult phenotypes of 5-weeks csn mutant plants, compared to the wild type plants of their corresponding ecotype backgrounds. csn5a-1, csn5a-2, csn5b-1, csn1-10, csn3-3 are in Col background, while csn2-5 is in Ler background. (B) Expression of CSN subunits in various tissues of Arabidopsis in wild type and csn5 mutants. Tissues were collected from wild type (Col-0) or csn5a2 or csn5b1 mutants as indicated. Total proteins were analyzed by immunoblotting using antibodies against CSN5A, CSN1, and CSN8. Anti-RPN6 blots on the same samples were used as an internal reference. csn5b-1 mutation had no detectable effect on the level of CSN subunits in the tissues examined. csn5a-2 mutation resulted in notable reduction in CSN5A level, and had minor effect on CSN1 and CSN8 levels. (JPG) [file pgen.1007237.s004.jpg]

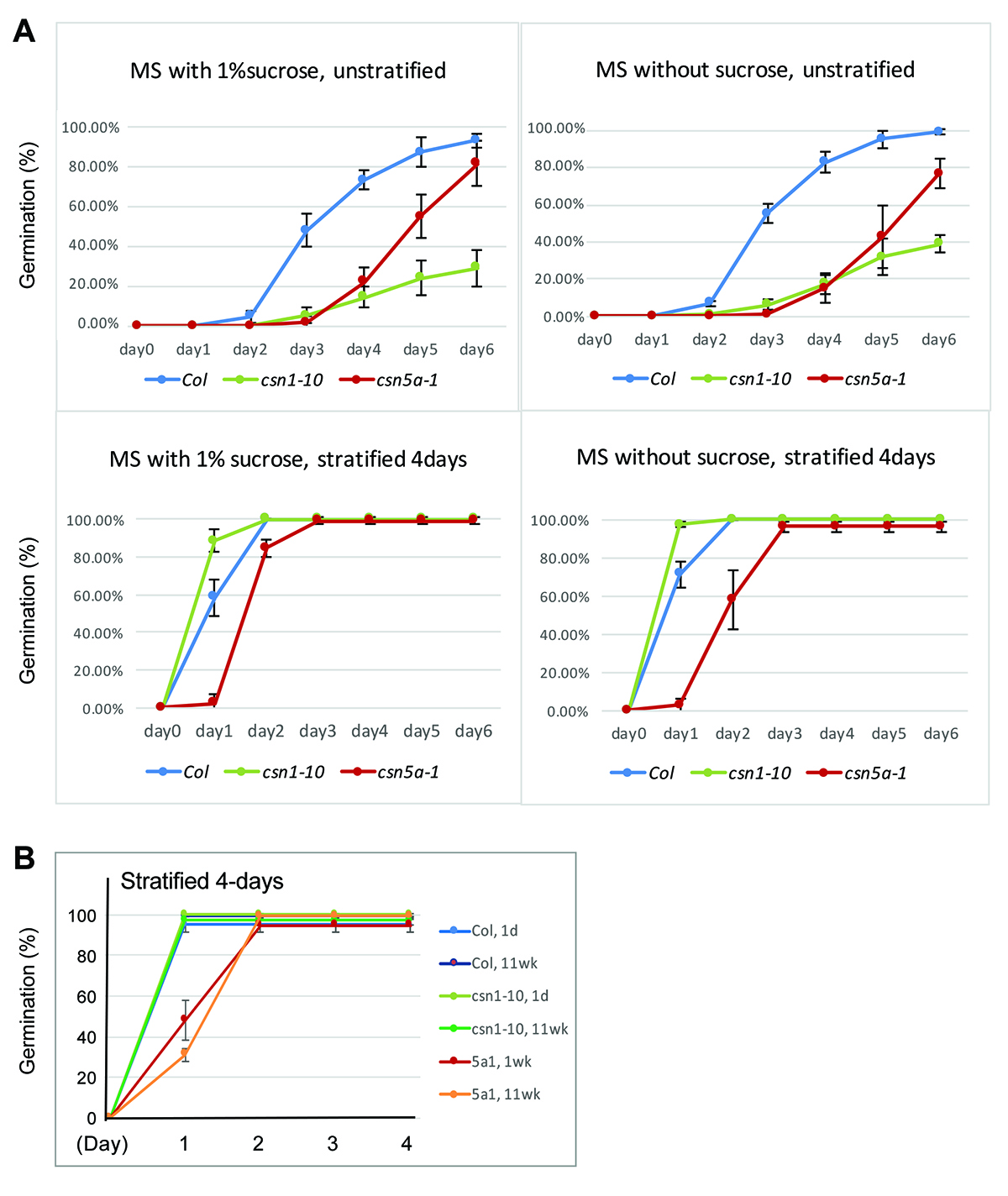

Supplement: S2 Fig — (A) Col, csn1-10 and csn5a-1 seeds (4-day after collection) were tested for germination on solid growth medium containing 1% sucrose (left panels), or had no sucrose (right two panels). Seeds were not stratified (top panels) or cold stratified for 4 days (bottom panels). The slow germination phenotype was clearly displayed on both sucrose-containing or sucrose-less plates. (B) Col and mutant seeds of indicated storage age were cold stratified for 4 days before the germination test. csn5a-1 showed delayed germination even after cold stratification of fully after-ripened seeds. (JPG) [file pgen.1007237.s005.jpg]

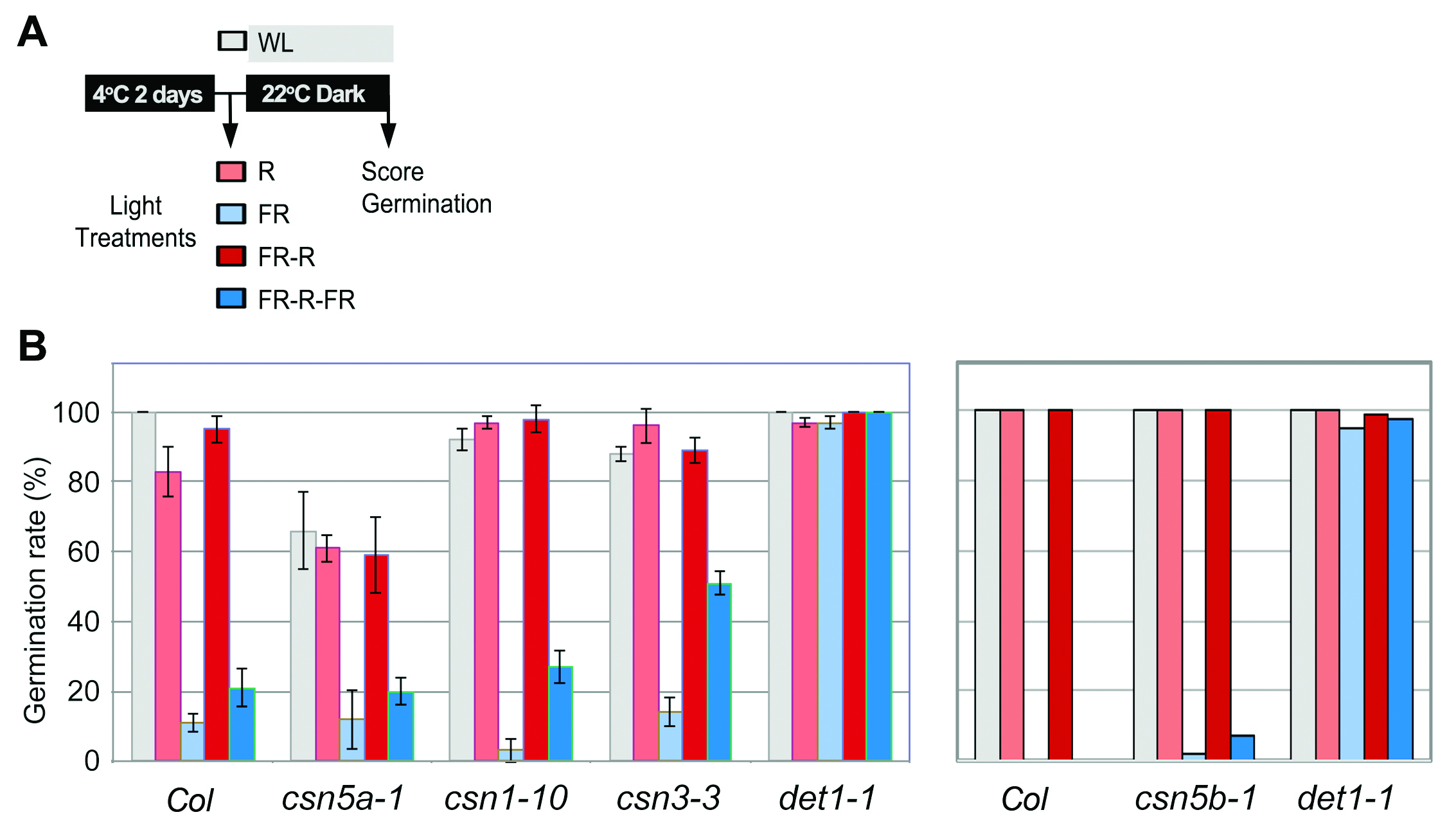

Supplement: S3 Fig — (A) The diagram illustrating the light treatment procedure used to test phyB-controlled seed germination. Seeds were cold stratified for 2 days prior to the light treatment. After light treatments, seeds were incubated in the dark at 22oC for two or three days. WL, constant white light; R, red light 5 min; FR, far-red light 5min; FR-R, far-red light 5min followed by red light 5 min; FR-R-FR, far-red light 5min followed by red light 5 min and followed by far-red light 5 min. (B) The germination rates at day-2 (or day-3 for csn5a-1) post light treatments are shown. The color code in (B) is identical to the colors indicating light treatment in (A). The csn mutants exhibited largely normal light responsive seed germination, while det1-1 (as a control) showed light-independent seed germination. Error bars represent standard deviation from 4 repeats. (JPG) [file pgen.1007237.s006.jpg]

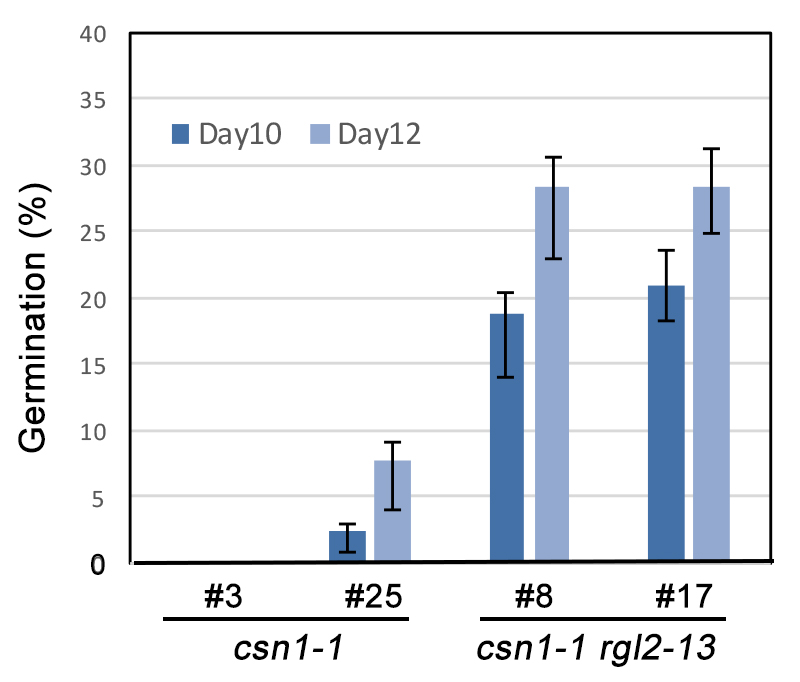

Supplement: S4 Fig — The graph shows the germination rates at day-10 and day-12 of two segregating sibling lines of csn1-1 and double mutant csn1-1 rgl2-13. Error bars represent standard deviation from 4 repeats. (JPG) [file pgen.1007237.s007.jpg]

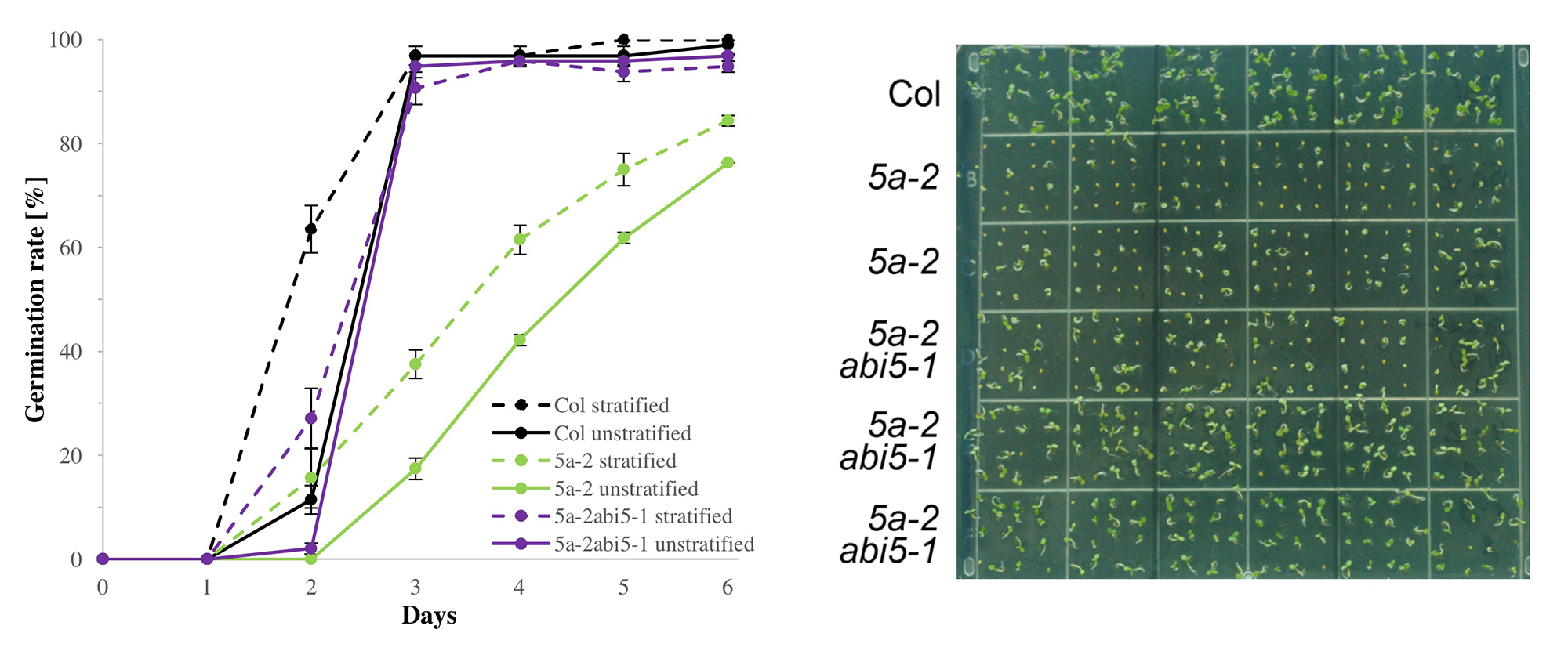

Supplement: S5 Fig — In the right panel, the csn5a-2 abi5-1 double mutants showed improved germination rates over csn5a-2. These mutant lines were segregating sibling lines from the same cross. Germination rate of one representative csn5a-2abi5-1 double and csn5a-2 single mutant lines were counted and graphed as shown on the left. Error bars represent standard deviation from 4 repeats. (JPG) [file pgen.1007237.s008.jpg]

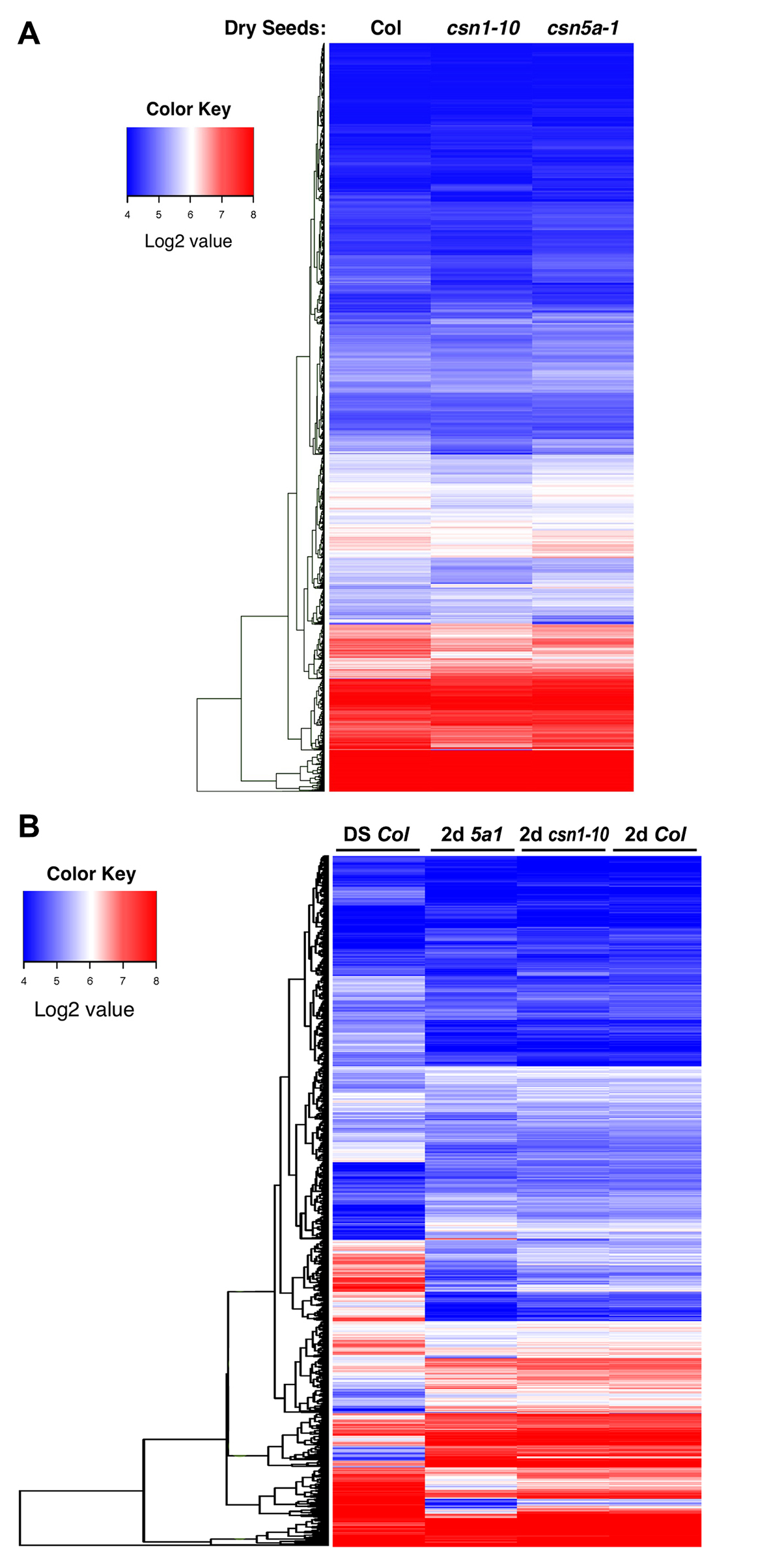

Supplement: S6 Fig — (A) Heatmap diagram of mRNA profiles in dry seeds of Col wild type, csn1-10, and csn5a-1. Genes with expression values from 13 and above were included. The color code is set from 13–300 in Log2 scale. Genes with expression level above 300 show the same color as those of 300 (red). The transcriptome profile of csn1-10 in dry seeds show greater dissimilarity to wild type than comparing csn5a-1 to wild type. (B) Heatmap plot showing gene expression profile in 2-day imbibed seeds of csn5a-1, csn1-10, and Col, compared to Col dry seeds. Genes with the expression value from 13 and above were included. The color code is set from 13 to 300 in Log2 scale. Genes with expression values above 300 are indicated with the same color as those of 300 (red). csn5a-1 displayed greater transcriptome changes than csn1-10 did at 2-day post imbibition. (JPG) [file pgen.1007237.s009.jpg]

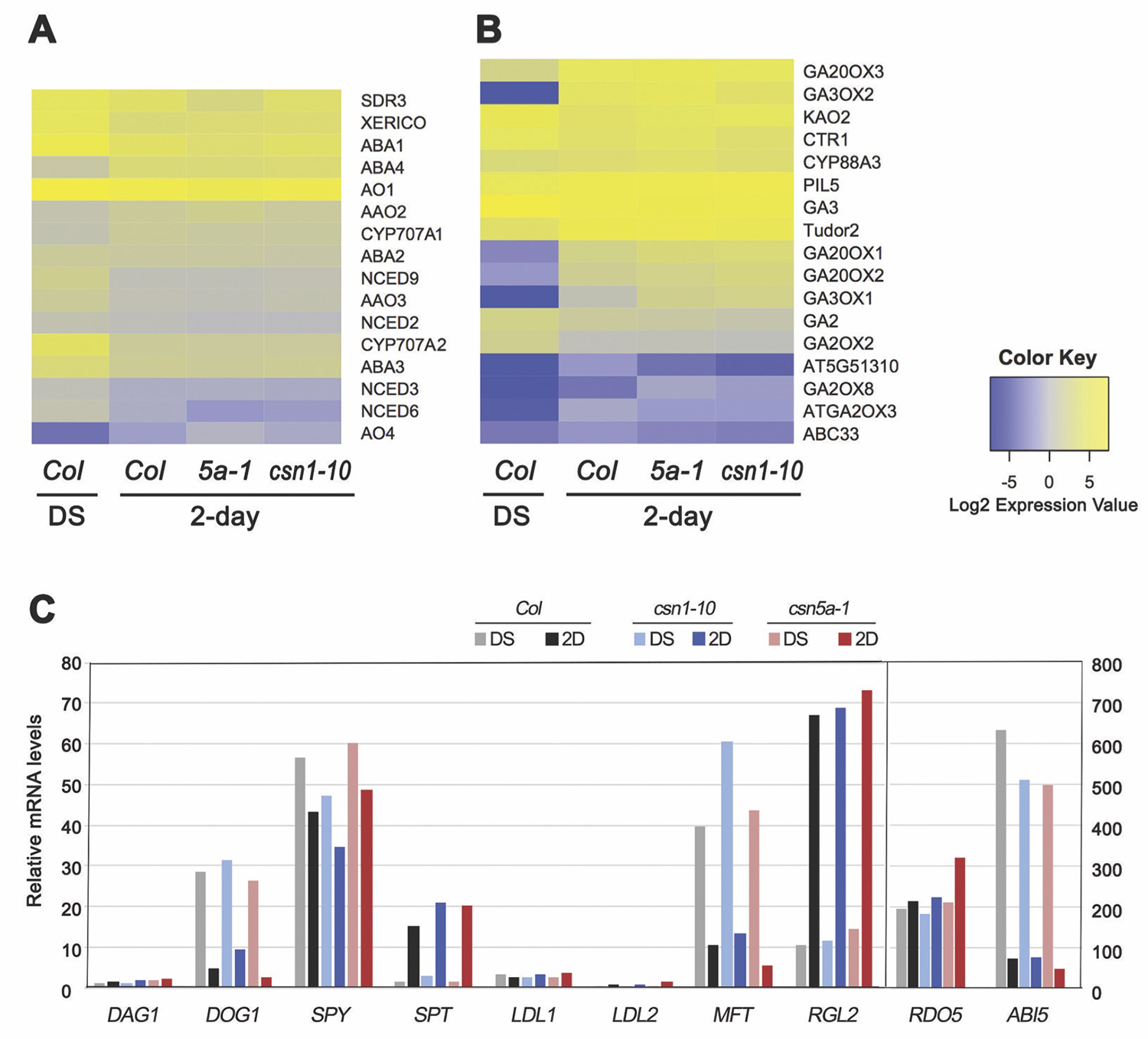

Supplement: S7 Fig — (A and B) Heatmap showing ABA-related genes (A) and GA related genes (B) in 2-day imbibed seeds of csn5a-1 (5a-1), csn1-10, and Col, compared to those of Col dry seeds. (C) A panel of known seed germination regulatory genes showed similar expression profiles in csn5a-1, csn1-10 and Col. These genes are: DAG1 (DOF AFFECTING GERMINATION 1 AT3G61850), DOG1 (DELAY OF GERMINATION 1, AT5G45830), SPY SPINDLY, AT3G11540), SPT SPATULA, AT4G36930), LDL1/2 (ARABIDOPSIS LYSINE-SPECIFIC HISTONE DEMETHYLAS AT1G62830/ AT3G13682), MFT (MOTHER OF FT AND TFL, AT1G18100), RGL2 (RGA-LIKE 2, AT3G03450), RDO5 (REDUCED DORMANCY 5, AT4G11040), ABI5 ABA INSENSITIVE 5, AT2G36270). (JPG) [file pgen.1007237.s010.jpg]
